# Overexpression of *Umellularia californica* FatB thioesterase affects plant growth and lipid metabolome leading to improved drought tolerance in *Arabidopsis* and tomato

**DOI:** 10.3389/fpls.2024.1446210

**Published:** 2025-01-10

**Authors:** Shuangshuang Wang, Biyun Yang, Yitao Liang, Xinrui Zou, Min Xu, Chan Zhao, Yiwen Wang, Bing Ni, Pinkuan Zhu, Yina Jiang

**Affiliations:** School of Life Sciences, East China Normal University, Shanghai, China

**Keywords:** medium chain fatty acid, FatB thioesterase, plant growth, drought tolerance, transcriptomic and lipid metabolomic analysis, methyl laurate, phosphatidylcholine

## Abstract

Frequent and extreme drought exerts profound effects on vegetation growth and production worldwide. It is imperative to identify key genes that regulate plant drought resistance and to investigate their underlying mechanisms of action. Long-chain fatty acids and their derivatives have been demonstrated to participate in various stages of plant growth and stress resistance; however, the effects of medium-chain fatty acids on related functions have not been thoroughly studied. Here, we integrate lipidomic, transcriptomic, and genetic analyses to elucidate the roles of the medium-chain acyl-acyl carrier protein thioesterase of *Umellularia californica* FatB (UcFatB) in drought tolerance and plant growth. *Arabidopsis* and tomato transgenic lines overexpressing *UcFatB* showed that the medium chain fatty acids mainly affect the male reproductive process of plant development. Transcriptomic and non-targeted lipid metabolomic combination analysis revealed significant changes in lauric acid-related metabolic pathways, as evidenced by increased phosphatidylcholine accumulation and upregulated stress-response gene expression. Consistent with the thicker waxy cutin layer and increased membrane integrity, *UcFatB-*overexpression enhanced drought tolerance in both *Arabidopsis* and tomato. Furthermore, methyl laurate and phosphatidylcholine application improved tomato drought resistance and fruit yield. These findings provide new insights into the potential genetic resources and cost-effective chemicals for enhancing drought resistance in crops.

## 1 Introduction

The global occurrence of drought is gradually increasing, with extreme drought greatly impacting vegetation growth and food production (Xu et al., 2019). Drought stress adversely affects agricultural productivity worldwide and is expected to increase in the future (Sharif et al., 2022). Drought stress exerts multiple effects on plant cells, including reduced turgidity, decreased photosynthetic activity, altered oxidative metabolism, and decreased membrane stability (Fahad et al., 2017). Additionally, it decreases leaf size, slows stem elongation, and reduces water-use efficiency (Alghabari et al., 2015; Alghabari et al., 2016). Tomato (*Solanum lycopersicum* L.), a vegetable crop with global importance, is susceptible to the adverse effects of drought stress. This environmental challenge poses a substantial threat, resulting in declines in tomato growth, yield, and fruit quality across multiple production regions (Steduto et al., 2012). Therefore, solutions to alleviate the effects of drought and to enhance the resistance to drought in tomato are critical.

Fatty acids (FAs) and their derivatives, which are essential for multiple biological functions, are organic components that serve as precursor molecules for lipids or substrates, which are essential for cellular energy production in all developing organisms (Papackova and Cahova, 2015). FAs are aliphatic chains that contain carboxylic acid functional groups. They can exist as either saturated or unsaturated FAs and can be categorized into different groups based on chain length: short-chain fatty acids that contain less than 6 carbon atoms, medium-chain fatty acids (MCFAs) possess 6–12 carbon atoms, long-chain fatty acids (LCFAs) contain more than 12 carbon atoms, and very long-chain fatty acids contain more than 20 carbon atoms. In plants, carbon chain elongation during *de novo* FA synthesis in plastids is terminated by the acyl-acyl carrier protein (ACP) thioesterase (FAT), which hydrolyzes the thioester bonds of acyl-ACPs to release free FAs and ACP (Bonaventure et al., 2003). It is well-known that oleate (18:1) and palmitate (16:0) are the primary products of plastid FA synthesis in *Arabidopsis* leaves. A large proportion of these products is exported as free FAs to the cytosol and subsequently re-esterified to form acyl-CoA. In eukaryotes, these compounds enter other pathways in other organelles, such as the endoplasmic reticulum, primarily for glycerolipid biosynthesis (Browse and Somerville, 2003). LCFAs (C16-C18) and their derivatives, including glycerides, phospholipids, sphingolipids and other lipids, have been demonstrated to participate in the cell endomembrane system as well as various stages of plant growth and development. These stages encompass seed germination, organ differentiation, male fertility, and fruit development (Boutté and Jaillais, 2020). In addition, they play crucial roles in regulating the resistance to biotic and abiotic stresses (Ali et al., 2018; Batsale et al., 2021; Hongyun and Yao, 2022).

In response to frequent drought perturbations, plants have evolved a variety of tolerance mechanisms, including changes in root system architecture to increase root water uptake and stomatal closure to reduce water loss and to adjust osmotic processes within cells, essentially maintaining physiological water balance (Gupta et al., 2020; Abboud et al., 2021). A previous study has shown that the response of plant cell membranes to drought stress involves heightened sensitivity to certain alterations, such as changes in saturated and unsaturated FA composition, FA oxidation, and phospholipid degradation (Armond et al., 1980; Quinn, 1988; Vígh et al., 1993). For instance, the cell membranes of apricot (*Armeniaca vulgaris* Lam.) leaf exhibit distinctive changes in response to drought stress, including a decrease in the content of linoleic acid, which is accompanied by increases in stearic acid, oleic acid, and linolenic acid levels (Yanping and Jiarui, 2002). Similarly, soybean (*Glycine max*) exhibits significant increases in saturated FA levels under drought stress, resulting in the peroxidation of a substantial amount of FAs and the formation of smaller carbon–hydrogen compounds, such as malondialdehyde, 4-hydroxynonenal, hydroxy FAs, ketone FAs, and their derivatives (Ramesh et al., 2020). In addition, the drought-induced accumulation of reactive oxygen species within cell membranes can target unsaturated FAs on the glycerol backbone, leading to increased FA oxidation and membrane permeability (Cruz de Carvalho, 2008; Lyublinskaya et al., 2015). In contrast to cell membranes, the thylakoid membranes in chloroplasts, which are mainly composed of glycolipids, serve as the site for the light-dependent reactions of photosynthesis, and their lipid components are highly sensitive to drought stress (Yuan et al., 2018). A previous study has also shown that drought stress induces significant decreases in monogalactosyl diacylgycerol, digalactosyl diacylglycerol, phosphatidylglycerol (PG), and chlorophyll contents within wheat (*Triticum aestivum*) thylakoid membranes (Zhao et al., 2005). Given the importance of membrane lipid composition, identifying the marker properties of membrane components, which affect membrane integrity and function, may be key to improving the ability of plants to cope with water scarcity in the future.

Studies in animals have shown that MCFAs (C6-C12) have unique effects on growth, reproduction, immunity, fat deposition, intestinal development, and intestinal flora. Consequently, they have wide applications in fields such as feed additives, food production, and medicinal products (Zentek et al., 2011; Vyas et al., 2012; Sun et al., 2013; Angenent et al., 2016). Some plant species that produce MCFAs possess an additional FatB enzyme that can catalyze medium-chain substrates such as C8:0/C10:0 (ChFatB2, *Cuphea hookeriana*) and C12:0 (UcFatB, *Umellularia californica*) (Voelker et al., 1992; Dehesh et al., 1996a; Thelen and Ohlrogge, 2002; Blatti et al., 2013). Due to the limited production of MCFAs by only a few plant species, genetic modifications to control FA chain length have gained considerable attention in previous studies. For example, the expression of *ChFatB2* in *Brassica napus* seeds improves the production of C8 and C10 FAs, and the introduction of *UcFatB* into *Arabidopsis* plants results in a 58% increase in laurate (C12:0) production (Dehesh et al., 1996b; Voelker et al., 1992). Despite these advances, the effects of MCFAs on plant growth, development, and related functions have not been thoroughly studied.

Here, through the overexpression of *UcFatB* in both *Arabidopsis* and tomato plants, we report that medium-chain FatB thioesterase is involved in various developmental processes. *UcFatB* overexpression in *Arabidopsis* compromised male fertility, seed growth, and leaf development. However, overexpression affected the weight and seed number per fruit in tomato plants. Surprisingly, *UcFatB* overexpression led to a strong drought resistance phenotype in both *Arabidopsis* and tomato plants. We demonstrate that *UcFatB* overexpression affects both the composition of lipid metabolites related to plant cell membranes and the expression of genes related to stress responses, and speculate that both are the main reasons for the enhanced plant drought tolerance. We also offer a potential application for phosphatidylcholine in improving tomato drought resistance and fruit yield. Taken collectively, these findings expand our understanding of the association between drought and vegetation and provide a potential strategy for enhancing drought tolerance through plant engineering.

## 2 Materials and methods

### 2.1 Plant growth and genotyping

*Arabidopsis* plants were grown in soil in greenhouse conditions (21℃) under long-day conditions (16-h of light/8-h of dark) or short-day conditions (8-h of light/16-h of dark). The *Arabidopsis* acyl-ACP thioesterase B (FatB; At1g08510) mutant *fatb-1* (SALK_020856) on a Col-0 background was obtained from the Nottingham Arabidopsis Stock Centre (NASC). The T-DNA insertion of *fatb-1* was verified using the T-DNA border primer SALKLB1.3 in combination with the gene-specific primers listed in **Supplementary Table 7**. All experiments used tomato cv. Micro-Tom (MT) as the wild type (control). Seeds were germinated on Murashige-Skoog (MS) medium and then transferred to soil and grown in greenhouse conditions (25°C) under a 16/8-h light/dark regime.

### 2.2 Molecular cloning and generation of transgenic plants

To generate lines overexpressing (OE) *UcFatB*, *Arabidopsis* and tomato plants were transformed with a *UcFatB* overexpression construct. For *UcFatB* overexpression analysis, the codon optimized synthetic *UcFatB* cDNA was cloned into *ProUBQ*-pK7WG2R. For *A.thaliana* complementation analysis, the *UBQ* promoter of *ProUBQ*-pK7WG2R was removed by HindIII and SpeI digestion and replaced with a 1.8kb genomic fragment of the *AtFatB* promoter, a clone containing the cDNA of *AtFatB* and *UcFatB* was each amplified by PCR and cloned into pENTR/SD/D-Topo. The fragment was then transferred from the entry vectors into pGWB601 by Gateway LR reactions. Primer sequences for plasmid construction are listed in **Supplementary Table 7**.

*UcFatB* OE Arabidopsis and tomato plants are produced using agrobacterium-mediated transformation (Clough and Bent, 1998). Transgenic plants were grown in 100 mM kanamycin (Sangon Biotech).

### 2.3 Quantitative real-time PCR analysis

Leaves of Col-0, Micro-Tom, and overexpressing transgenic plants were collected, and total RNA was extracted using a Trizol-based (Invitrogen) method to analyze the expression levels of *UcFatB* and other downstream genes. After DNase I digestion and first-strand cDNA synthesis, quantitative real-time PCR was performed with SYBR premix Ex Taq II (Takara) on a CFX Connect real-time system (Bio-Rad) using the primers listed in **Supplementary Table 7.**

### 2.4 Measurement of seed mass and microscopic analysis of embryos

One thousand seeds from Col-0, UBQ, and *UcFatB* OE transgenic lines were randomly counted and weighed using a microbalance. The size of the seeds was determined by measuring the length and width using Image J software, and whole seeds were observed under a Zeiss Axio Imager A2 light microscope (Germany).

### 2.5 Phenotypic analysis of flowers and anthers

Plants were photographed using a digital camera (Canon, Japan). *Arabidopsis* flower images were captured under a Zeiss Axio Imager A2 light microscope (Oberkochen, Germany). Stage 12 anthers were collected and stained 15 minutes at 65°C using an Alexander solution prepared following published protocols (Alexander, 1969). Anthers were pressed to release the stained pollen grains and photographed using a Zeiss Axio Imager A2 light microscope (Germany).

### 2.6 Electron microscopy observation

Seeds and fresh pollen grains released from stage 13 anthers were coated with gold and observed under a JSM-6360LV scanning electron microscope (Tokyo, Japan). For transmission electron microscopy (TEM) observation, the leaves were fixed in glutaric dialdehyde buffer and embedded in freshly mixed resin. Ultrathin sections (80 nm thick) were observed using a Tecnai G2 F30 TEM (FEI, Czech Republic).

### 2.7 Drought stress treatments

For drought stress treatments, *Arabidopsis* seeds were germinated on full-strength 1/2 MS medium and then transferred to the soil after two weeks, and seedlings were cultivated in the greenhouse at 21°C under a 16/8-h light/dark regime. Tomato seeds were germinated on Murashige-Skoog (MS) medium. Seedlings were then transferred to soil and grown in the greenhouse at 25°C under a 16/8-h light/dark regime. Arabidopsis plants grown in soil were well irrigated for four weeks, and tomato plants grown in soil were well irrigated for six weeks after germination before drought stress treatments. In each experiment, 15 plants per genotype were used for the treatment. Drought stress was induced by withholding water for 28 days. Control plants were watered throughout the experiment. Mannitol-mediated drought stress was induced by transferring 7-day-old tomato seedlings to 1/2 MS plates containing 100 mM mannitol for 10 days. All stress treatment experiments were performed in three independent trials.

### 2.8 Relative water content

Terminal leaflets were harvested and immediately weighed to determine their fresh weight (FW). Subsequently, leaves were immersed in distilled water and incubated at 4°C overnight to obtain the saturated weight (SW). Leaves were then dried at 60°C for 48 hours to measure the dry weight (DW). RWC was calculated using the formula RWC % = ((FW−DW)/(SW−DW)) × 100%.

### 2.9 Water loss

Detached tomato terminal leaflets were placed in a growth chamber at 25°C. The leaf weight was recorded at the time points indicated in the figures and expressed as a percentage of the initial fresh weight.

### 2.10 RNA sequencing and data analysis

Rosettes from 4-week-old Col-0 and *UC16* (abbreviation for *UcFatB*-overexpression line 16) transgenic plants were collected and immediately frozen in liquid nitrogen. Total RNA was extracted from each sample using the TRIzol-based method (Invitrogen). RNA libraries were sequenced on an Illumina Novaseq 6000 platform by OE Biotech, Inc. (Shanghai, China). Data were deposited with the National Center for Biotechnology Information under Submission ID: SUB13799737 and BioProject ID: PRJNA1009883).

### 2.11 Metabolomics analysis

Total lipids from 4-week-old *Arabidopsis* rosette leaves (*UC16* vs. Col-0) were extracted and analyzed using an ultra-high-performance liquid chromatography (UHPLC) system fitted with a Q-Exactive quadrupole-Orbitrap mass spectrometer (MS) equipped with a heated electrospray ionization (hESI) source and used to analyze metabolic profiles in both ESI positive and negative ion modes. The original LC-MS data were processed using Progenesis QI V2.3 (Nonlinear, Dynamics, Newcastle, UK) for baseline filtering, peak identification, integration, retention time correction, peak alignment, and normalization. Compound identification was based on the precise mass-to-charge ratio (M/z), secondary fragments, and isotopic distribution, using the Human Metabolome Database (HMDB), LipidMaps (V2.3), Metlin, EMDB, PMDB, and self-built databases for qualitative analysis.

### 2.12 Statistical analysis

In the untargeted metabolite-transcript correlation analysis, the top 20 items with the greatest difference in gene significance (log2(FoldChange)) and the top 20 items with the greatest difference in metabolite significance (log2(FoldChange)) were selected to calculate the correlation between them. Pearson`s correlation coefficients were calculated between the metabolome and transcriptome data. *P < 0.05, **P < 0.01, ***P < 0.001.

Other experimental data were statistically analysed using Prism 9.0 Software (GraphPad, USA). Mean ± SE are shown in the statistical figures. Statistically significant differences between control and experimental groups were determined by Two-sided Students’ t-test (*P < 0.05; **P < 0.01; *ns*, not significant). The *P*-value used by ANOVA analysis (*P* < 0.05 or *P* < 0.01) is indicated in the legends and different letters indicate significant differences.

### 2.13 Accession numbers

Sequence data from this article can be found in the Arabidopsis Genome Initiative database under the following accession numbers: *FatB* (At1g08510), *ACTIN2* (AT3G18780), *PP2A* (At1g59830). Germplasm used included *fatb-1* (SALK_020856).

## 3 Results

### 3.1 UcFatB participates in a unique lipid metabolism pathway and affects *Arabidopsis* seed development

Although the substrate binding pockets and catalytic residue domains of the predicted amino acid sequences of acyl-ACP thioesterase B proteins from *Arabidopsis* (AtFatB) and tomato (SlFatB) aligned well with UcFatB, the C-terminal and N-terminal tails were more varied (**Supplementary Figure 1**). In addition, UcFatB and CcFatB (*Cinnamomum camphora*) formed a distinct clade in the acyl-ACP thioesterase B gene family (**Supplementary Figure 2**), indicating that the divergent regions have distinct structural roles. *UcFatB* may encode a unique lipid acyl-ACP thioesterase that differs from the well-characterized FatB proteins.

**Figure 1:**
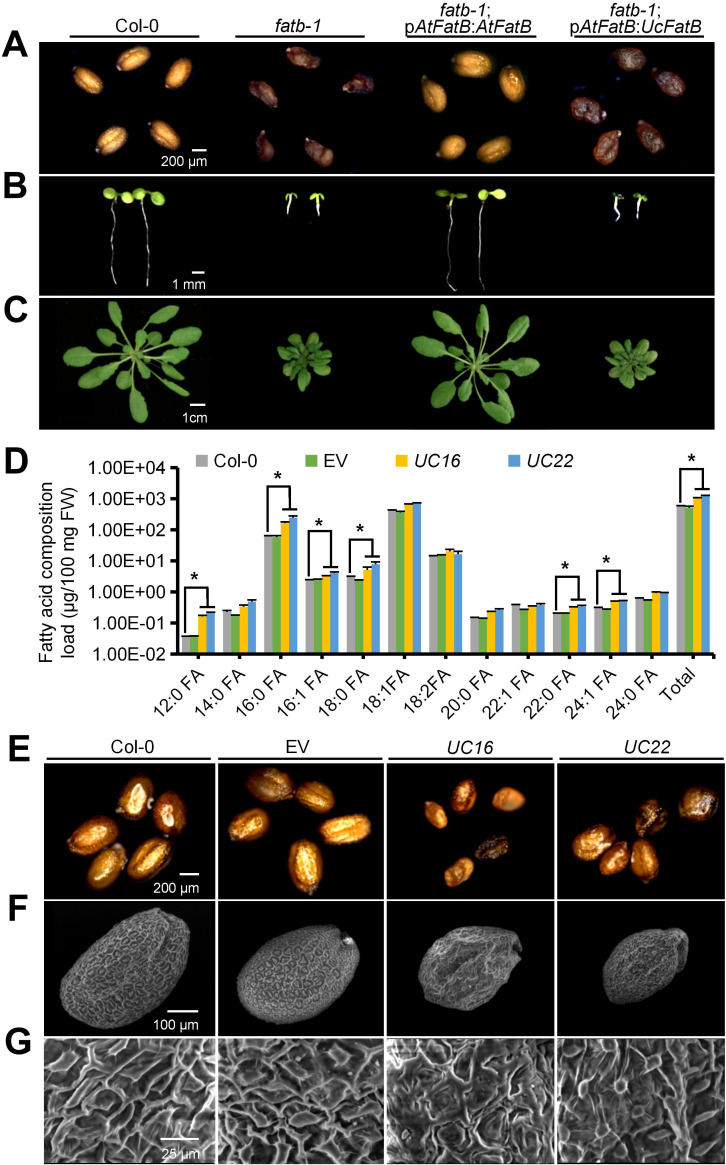
Generation of transgenic *Arabidopsis* lines overexpressing *UcFatB*. **(A-C)** Morphologies of mature seeds **(A)**, 7-day-old seedlings **(B)**, and 4-week-old rosette leaves **(C)** from *Arabidopsis* Col-0, *fatb-1* (SALK_020856), *fatb-1*; p*AtFatB: AtFatB* (*fatb-1* transformed with *AtFatB* cDNA driven by its native promoter), and *fatb-1*; p*AtFatB: UcFatB* (*fatb-1* transformed with *UcFatB* codon-optimized cDNA driven by *AtFatB* promoter) transgenic plants. **(D)** Fatty acid composition of 4-week-old rosette leaves in Col-0, empty vector control (EV), and two independent *UcFatB* overexpressing (*UcFatB-*OE) transgenic lines (*UC16* and *UC22*). FW, fresh weight. Error bars represent mean ± standard error of three biological replicates from 20 independent plants. Two-tailed Student’s t-test, compared to Col-0, *P < 0.05. **(E)** Morphologies of mature seeds from Col-0, EV, *UC16*, and *UC22* transgenic plants. **(F, G)** Scanning electron microscopy observations of the surface of Col-0, EV, *UC16*, and *UC22* transgenic plant seeds. Scale bars represent: 200 μm **(A)**, 1 mm **(B)**, 1 cm **(C)**, 200 μm **(E)**, 100 μm **(F)**, 25 μm **(G)**.

In *Arabidopsis*, the *fatb-1* (SALK_020856) mutant, which showed a significant reduction in *AtFatB* expression, exhibited a 50% decrease in palmitate (16:0) in free FAs across almost all tissues, including leaves, roots, flowers, and seeds, as well as a 30% decrease in stearate (18:0) in seeds. Consequently, the *fatb-1* mutant displayed a reduced growth rate, fresh weight loss, wrinkled seeds, and a decreased total wax load in leaves and stems (Bonaventure et al., 2003; Jiang et al., 2018). To confirm the functional difference between UcFatB and AtFatB, we fused the *AtFatB* promoter to the artificial codon-optimized cDNA sequence of *UcFatB* (Q41635.1) and introduced the resulting transgene into the *Arabidopsis fatb-1* mutant. While the *fatb-1* phenotype was restored to the wild-type by introducing a transgene of *AtFatB* driven by its native promoter (*fatb-1*; p*AtFatB*: *AtFatB*), the *fatb-1*; p*AtFatB*: *UcFatB* transgenic lines did not display any noticeable phenotypic variations compared to the *fatb-1* plants (**Figures 1A–C**; **Supplementary Figures 3A, B, D**). These findings indicate that the expression of *UcFatB* is insufficient to rescue the impaired development phenotype of *fatb-1* and imply that *UcFatB* has distinct roles in plant development compared to *AtFatB*.

In a previous study, we overexpressed the codon-optimized *UcFatB* cDNA in Col-0 and successfully generated *UcFatB*-overexpression (OE) homozygous T3-generation *Arabidopsis* lines, which were designated as *UC16* and *UC22*, respectively (**Supplementary Figure 3C**) (Jiang et al., 2017). Although earlier findings indicated that the overexpression of *UcFatB* fused with the *Brassica rapa* seed storage protein (napin) promoter caused a significant increase in MCFA (C12:0, C14:0) production and no significant changes in the number, size, or viability of *Arabidopsis* seeds (Voelker et al., 1992), we observed changes in the contents of most FA components in 4-week-old rosette leaves of *UcFatB*-OE transgenic lines (*UC16* and *UC22*) compared to the wild-type plants. Notably, the composition of MCFAs (C12:0), LCFAs (C16:0/16:1/18:0), and VLCFAs (C22:0/24:1) showed significant increases in *UC16* and *UC22* (**Figure 1D**), suggesting that *UcFatB* overexpression contributes to the accumulation of medium- and long-chain FAs in *Arabidopsis* rosette leaves.

Furthermore, we observed morphological changes in the *UC16* and *UC22* seeds. Specifically, the seeds of *UcFatB* overexpression lines were visibly smaller, displayed a darker seed coat color, and exhibited deformities in seed morphology, compared to those of the wild-type plants (**Figure 1E**), which resulted in significantly reduced seed weight (13.9 ± 0.1 for *UC16* and 15.1 ± 0.1 for *UC22* compared to 20.8 ± 0.1 μg/seed of the wild-type) and daily germination rate compared to the wild-type (**Supplementary Figures 3E, G**). Closer examination by scanning electron microscopy (SEM) showed that mature dry seeds of the *UcFatB-*OE plants had multiple deformities in seed morphology, such as wrinkled surfaces and irregular epidermal cells with fewer columella heaps, whereas wild-type seeds had smooth, dome-like epidermal cells (**Figures 1F, G**). These observations suggest that the disturbance of lipid accumulation caused by the expression of *UcFatB* can affect seed development.

### 3.2 *UcFatB-*OE plants are deficient in pollen exine formation, cell size, and chloroplast development

**Figure 2:**
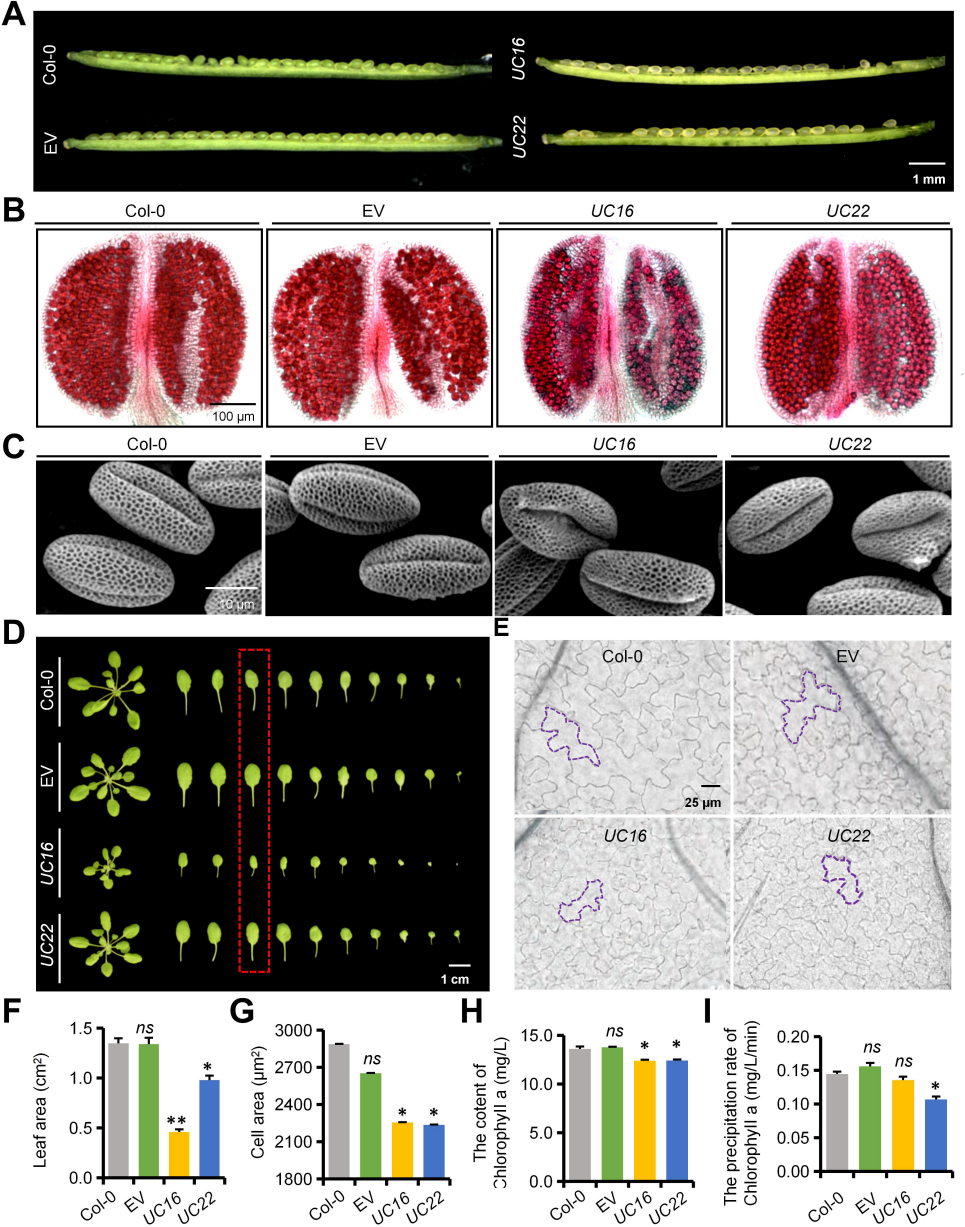
Phenotypic analyses of siliques, anthers, pollens and leaves of the wild-type, empty vector control (EV), *UC16*, and *UC22* plants. **(A-C)** Phenotypic analyses of immature silique **(A)**, anther **(B)**, and pollen exine **(C)** from 6-week-old Col-0, EV, *UC16*, and *UC22* transgenic plants. **(D, E)** Phenotypic analyses of single leaf **(D)** and leaf cell **(E)** using the epidermis of the adaxial parts of 4-week-old Col-0, EV, *UC16*, and *UC22* transgenic plant rosette leaves. **(F-H)** Quantification of leaf area **(F)**, cell area **(G)**, and Chlorophyll a content for indicated genotypes corresponding to **(D)** in red dashed box. **(I)** The precipitation rate of Chlorophyll a leaching for indicated genotypes corresponding to **(D)** in red dashed box. Error bars in **(F, G)** represent mean ± standard deviation of 30 biological replicates from five independent plants. Error bars in **(H)** and **(I)** represent mean ± standard error of three biological replicates from 20 independent plants. Two-tailed Student’s t-test, compared to Col-0, *P < 0.05, **P < 0.01. *ns*, statistically nonsignificant. Scale bars represent 1 mm **(A)**, 100 μm **(B)**, 10 μm **(C)**, 1 cm **(D)**, and 25 μm **(E)**.

To further investigate the underlying reasons for the differences in *UC16* and *UC22* seed development, we observed the phenotype throughout reproductive development from flowering onwards. *UC16* and *UC22* plants displayed normal floral development, and anthers had bulbous yellow surfaces (**Supplementary Figure 4A**). By contrast, microscopy revealed that the *UcFatB*-OE plants produced anthers that contained fewer viable pollen grains compared to the wild-type plants (**Figure 2B**), and scanning electron microscopy (SEM) showed that the outer wall structure of *UcFatB*-OE microspores was fuzzy compared to the well-organized pollen exine structure of the wild-type microspores (**Figure 2C**). Consequently, the siliques of *UC16* and *UC22* occasionally failed to produce enough mature seeds (**Figure 2A**; **Supplementary Figures 4B–E**). Furthermore, qRT-PCR analysis indicated that *UcFatB* overexpression led to a significant increase in the expression levels of genes related to lipid acyl metabolism and pollen exine formation, including *A6* (Hird et al., 1993), *ARABIDOPSIS THALIANA ANTHER 7* (*ATA7*) (Rubinelli et al., 1998), *LESS ADHESIVE POLLEN 5* (*LAP5*) (Dobritsa et al., 2010), *CYP703A2* (Morant et al., 2007), and *ACYL-COA SYNTHETASE 5* (*ACOS5*) (Souza et al., 2009). However, the expression levels of tapetum marker genes, including *DYSFUNCTIONAL TAPETUM1* (*DYT1*) (Zhang et al., 2006) and *MYB103* (Higginson et al., 2003), remained unchanged in *UcFatB*-OE plants (**Supplementary Figure 4F**). These findings suggest that *UcFatB* may disrupt pollen exine formation by promoting the synthesis of sporopollenin, a lipid-based polymer, thereby affecting male fertility and impacting seed development.

We examined whether the nutritional growth of *UcFatB-*OE plants differs from that of wild-type plants. We found that 5-day-old *UcFatB-*OE seedlings displayed stunted primary roots and decreased hypocotyl length compared to wild-type seedlings (**Supplementary Figures 5A–F**). Over the initial 4 weeks of growth under short-day conditions, the rosettes of *UcFatB-*OE plants exhibited an obvious reduction in diameter compared to wild-type rosettes, primarily due to decreased petiole length and leaf area (**Figures 2D, F**; **Supplementary Figures 5G, H**). We then compared cell areas in the epidermis of the adaxial parts of the eighth leaf from the bottom between the *UcFatB-*OE and wild-type seedlings and found that epidermal cells of *UC16* and *UC22* leaves were notably smaller compared to the wild-type, indicating that a reduction in cell size may be the primary factor contributing to the decreased leaf area (**Figures 2E, G**). Moreover, we observed that the leaf color of *UcFatB*-OE plants was lighter than that of wild-type plants throughout development, which may be linked to a reduction in chlorophyll content per equivalent fresh weight. Correspondingly, *UcFatB* overexpression resulted in a significant decrease in chlorophyll a content in the rosette leaves compared to the wild-type, whereas no apparent change was observed in chlorophyll b content (**Figure 2H**; **Supplementary Figure 5I**). Thus, the pale green phenotype observed in the *UcFatB*-OE transgenic plants is directly associated with the reduction in chlorophyll content.

### 3.3 Selective changes of lipid metabolome components in *UcFatB*-OE transgenic plants

Our previous data indicated that *UcFatB* overexpression leads to changes in half of the 12 FA components (C12-C24) in *Arabidopsis* leaves. Phenotypic changes during nutrition and reproductive development, including alterations in pollen exine structure and chlorophyll content, are also associated with lipid metabolism (Zhang et al., 2016; Hölzl and Dörmann, 2019). To further understand the effects of *UcFatB* overexpression on lipid metabolism and to analyze how changes in FA composition and content affect lipid synthesis, we performed LC-MS analysis on 4-week-old *UC16* and wild-type Col-0 rosette leaves. Principal component analysis (PCA) showed good repeatability, with all four biological replicates of wild-type Col-0 clustering separately with *UC16*, clearly distinguishing the two genotypes (**Supplementary Figure 6A**). A total of 569 species in 24 common classes of lipids were validated in *UC16* and Col-0 leaves and used for lipidomic analyses, and the ratio of each lipid species to the total lipid species was calculated; these included 70 species of fatty acyls, 158 species of glycerolipids (GLs, including DG, MG, TG, MGDG, SQDG, SQMG), and 3 major lipid classes in plant cell membranes, namely glycerophospholipids (GPLs, 246 species), sphingolipids (SLs, 72 species), and sterols (STs, 23 species) (**Supplementary Figure 6B**; **Supplementary Table 1**).

**Figure 3:**
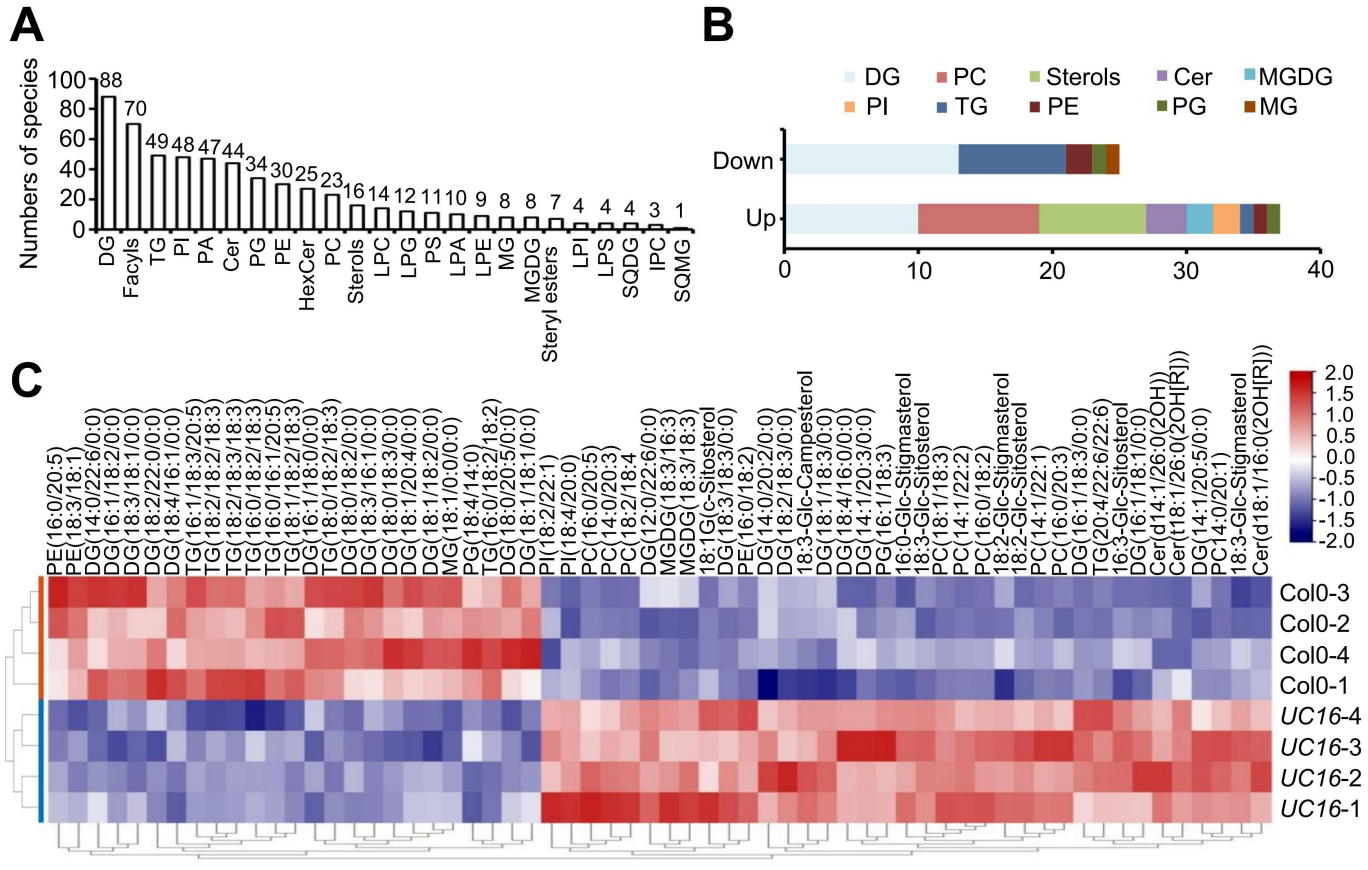
Lipid profiles of the leaf tissues of Col-0 and *UC16* transgenic plants. **(A)** Distribution of the total lipid species detected in 4-week-old Col-0 and *UC16* transgenic plant rosette leaves. The number of species in each lipid group is indicated. **(B)** Distribution of differentially (up and down) lipid species in *UC16* transgenic plant rosette leaves compared to Col-0. **(C)** Heatmap of 62 differentially produced lipid components in 4-week-old Col-0 and *UC16* transgenic plant rosette leaves. The name and abbreviation of lipid species in **(A, B)** are Diglyceride (DG); Triglyceride (TG); Phosphatidylinositol (PI); Phosphatidic acid (PA); Ceramide (Cer); Phosphatidylglycerol (PG); Phosphatidylethanolamine (PE); Hexosyl ceramide (HexCer); Phosphatidylcholine (PC); Lyso-PC (LPC); Lyso-PG (LPG); Phosphatidylserine (PS); Lyso-PA (LPA); Lyso-PE (LPE); Monoglyceride (MG); Monogalactosyl diacylglycerol (MGDG); Lyso-PI (LPI); Lyso-PS (LPS); Sulfoquinovosyl diacylglycerol (SQDG); Ceramide phosphoinositols (IPC); Sulfoquinovosyl monoacylglycerol (SQMG).

In our analysis, diacylglycerol (DG) contained 88 species (~15.36% of total) and was the most abundant class of lipids identified. DG serves as the structural backbone of GPLs, which differ from each other by their head groups. We identified phosphate in phosphatidic acids (PAs), choline in phosphatidylcholines (PCs), ethanolamine in phosphatidylethanolamines (PEs), glycerol in phosphatidylglycerols (PGs), inositol in phosphatidylinositols (PIs), and serine in phosphatidylserines (PSs) (**Figure 3A**), which was consistent with previous studies (Fahy et al., 2005; van Meer et al., 2008). Fatty acyls were the second detected class (~10.52% of total), and they included free FAs and the lipid chain sources of SLs. SLs include ceramides (Cers), which contain a sphingoid long-chain base connected to an FA chain as the core structure, and they can be classified as simple sphingolipids (Cers), glucosylceramides (GlcCers, including HexCer), and phosphosphingolipids (PCers, including IPC) based on the head group (**Supplementary Table 1**).

Quantitative metabolomic profiling via non-targeted lipid scanning revealed significant and selective changes in metabolite levels, including 37 significantly up-regulated and 25 significantly down-regulated products in *UC16* compared to wild-type Col-0 (**Figure 3B**; **Supplementary Table 2**). DG (10/13, up/down) accounted for 37% of the total significantly regulated products, resulting in no significant difference in the total load of DGs between *UC16* and wild-type Col-0. Furthermore, there were 8 species of TGs among the total down-regulated metabolites, whereas there were 9 species of PCs containing unsaturated acyl chains among the up-regulated metabolites, 4 of which had C14 acyl chains (**Supplementary Table 2**). Finally, the total load of TGs decreased to ~69%, and the total load of PCs was increased by ~2.66-fold in *UC16* compared to wild-type Col-0 (**Figure 3C**; **Supplementary Table 2**). Further analysis revealed that 4 species of PC (14:0/20:1, 14:0/20:3, 16:0/20:3, 16:0/20:5) were enriched in Kyoto Encyclopedia of Genes and Genomes (KEGG) metabolic pathways and classified as part of linoleic acid metabolism (ath00591), alpha-linolenic acid metabolism (ath00592), and glycerophospholipid metabolism (ath00564) (**Supplementary Tables 3**, **4**; **Supplementary Figure 9**).

### 3.4 Untargeted metabolite-transcript correlation reveals a candidate gene cluster responsible for stress resistance

To further elucidate the molecular mechanisms underlying the phenotypes *UcFatB* overexpression, we conducted transcriptome analyses on 4-week-old rosette leaves of the wild-type and *UC16* plants using 3 biological replicates. PCA showed good repeatability, with the biological replicates of Col-0 and *UC16* clustering together and showing clear separations between Col-0 and *UC16* materials (**Supplementary Figure 7A**). An overview showed the patterns of 3196 differentially expressed genes (DEGs) with *P* < 0.05 and fold change > 2 or < 0.5 between wild-type and UC16 (**Supplementary Figure 8B**; **Supplementary Table 5**). Among these DEGs, 1451 were up-regulated and 1745 were down-regulated in *UC16* compared to the wild-type (**Supplementary Figure 7C**).

**Figure 4:**
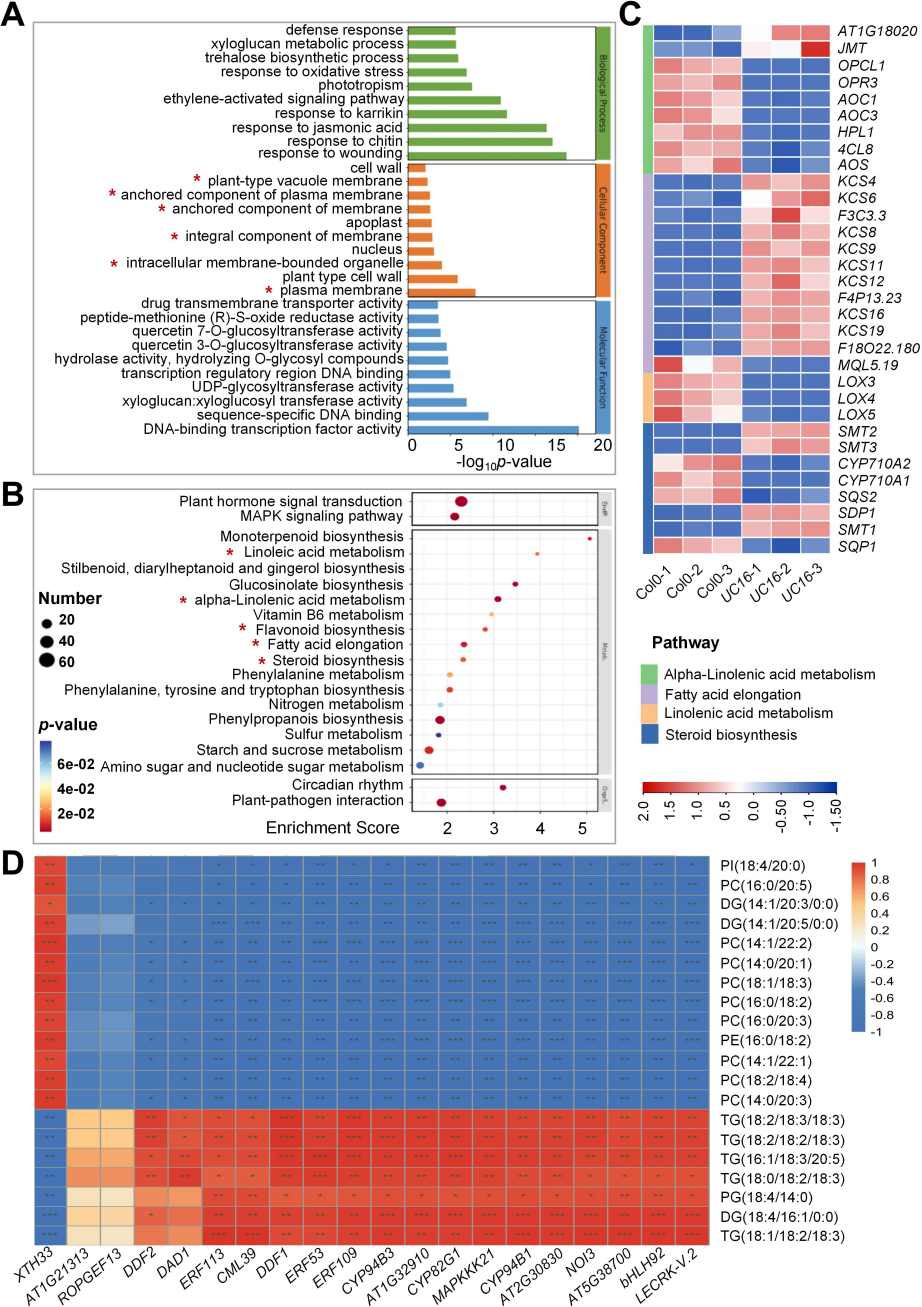
Untargeted metabolite-transcript correlative combination analyses of Col-0 and *UC16*. **(A)** Top 30 Gene ontology (GO) enrichment terms of genes with changed expression in *UC16* leaves compared to Col-0. **(B)** Top 20 KEGG enrichment terms of genes with changed expression in *UC16* leaves compared to Col-0. **(C)** Heatmap of fatty acid elongation, steroid biosynthesis, alpha-linolenic acid metabolism, and linolenic acid metabolism-related differentially expressed genes. The color displays the fold change. Red indicates upregulation in gene expression, and blue indicates downregulation. The darker in color, the greater fold change. **(D)** Integrated metabolome and transcriptome analyses. Pearson’s correlation coefficients were calculated between the metabolome and transcriptome data. *P < 0.05, **P < 0.01, ***P < 0.001.

To analyze the potential functions of *UcFatB*-OE responsive genes, DEGs were subjected to Gene Ontology enrichment analyses. Among the top 30 enriched terms of 3 categories, the DEGs were significantly enriched in different membrane components in the cellular component category and responses to different stresses/phytohormones in the biological process category (**Figure 4A**). Consistent with the metabolomic analysis, the DEGs were significantly enriched in “linolenic acid metabolism”, “alpha-linolenic acid metabolism”, “fatty acid elongation”, and “steroid biosynthesis”, and they were among the top 20 significantly enriched metabolism-related processes (*P* < 0.5) in KEGG enrichment analysis (**Figures 4B, C**). To validate the transcriptome data, 8 of these genes were randomly selected for qRT-PCR analysis (**Supplementary Figure 8**), and their expression levels were consistent with the RNA-seq data.

We used combination analysis to correlate the untargeted metabolomic data with the RNA-seq data to describe the relationships between metabolites and genes (**Supplementary Table 6**). Pearson correlation analyses were conducted on the top 20 DEGs and the top 20 differentially accumulated metabolites (DAMs) to reveal the functional relationships and to identify new regulatory mechanisms related to lipid metabolism. The 20 DEGs showed strong positive and negative correlation coefficient values (R2 > 0.8 or < -0.8 and *P* < 0.05) with the 20 metabolites. For example, there was a significantly negative correlation between the expression levels of *LECRK-V.2*, *BHLH92*, *AT5G38700*, and *NOI3* genes with the metabolite abundances of PCs, PIs, and PEs, whereas *XTH33* expression was positively correlated with the levels of these metabolites (**Figure 4D**).

Further analysis indicated that most of the top 20 DEGs were related to stress responses. For example, *NOI3*, *CYP94B1*, *ERF109*, and *CML39* were significantly positively related to salt stress responses (Vanderbeld and Snedden, 2007; Renault et al., 2013; Bahieldin et al., 2016; Krishnamurthy et al., 2020). Moreover, the mRNA expression level of *ERF113*, which is negatively correlated with drought tolerance (Zhu et al., 2020), was found to be significantly down-regulated in *UC16* (**Supplementary Table 5**), suggesting that overexpression of *UcFatB* may enhance drought tolerance in plants.

### 3.5 *UcFatB* overexpression improves drought tolerance in *Arabidopsis*

**Figure 5:**
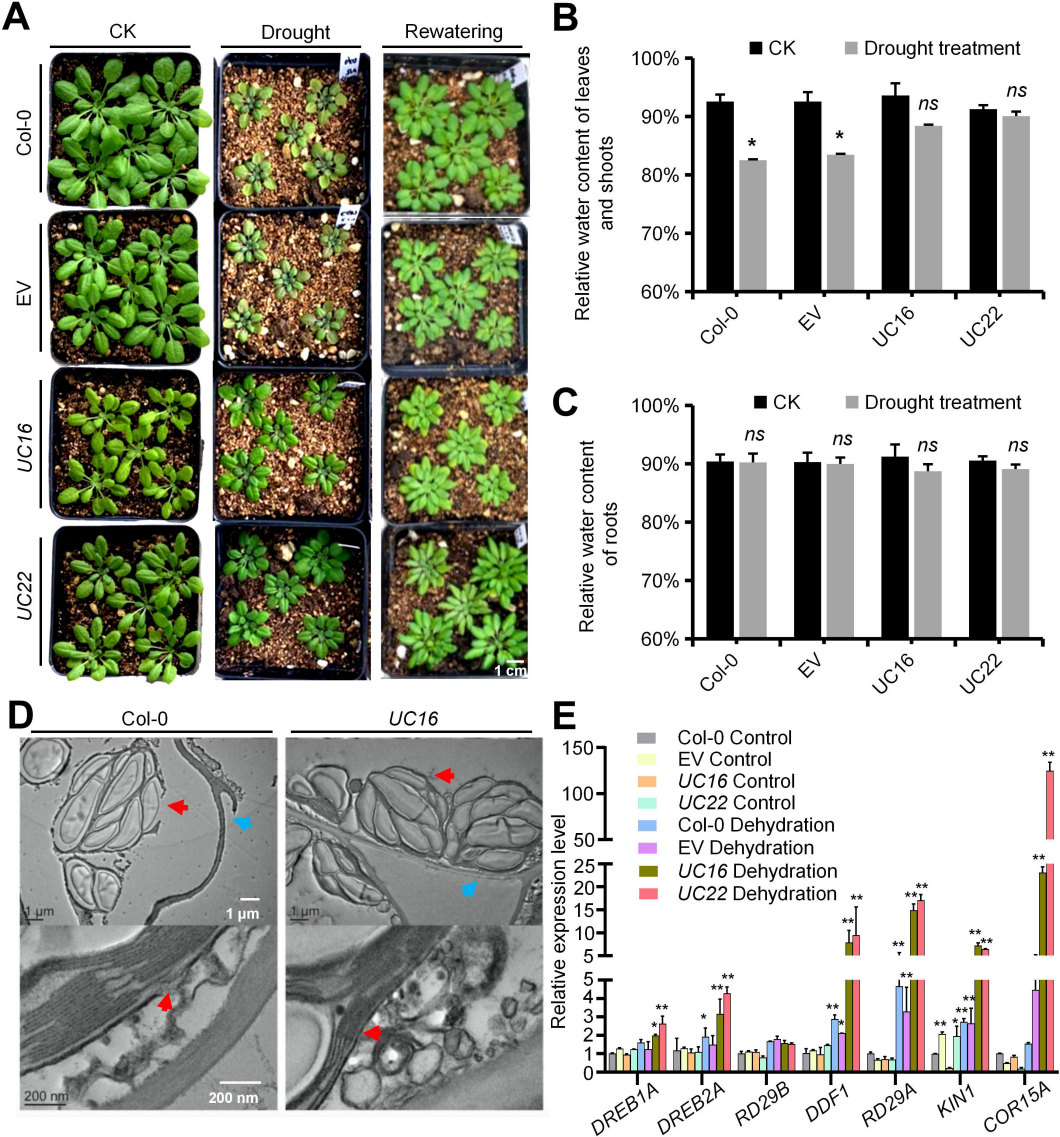
*UcFatB* OE transgenic plants with obviously improved drought tolerance. **(A)** *Arabidopsis* drought tolerance assay. Col-0, EV, *UC16*, and *UC22* plants grown under short-day conditions before drought stress was induced. Photos were taken at the beginning of rewatering (just after the 2-week drought stress) and one week after rewatering. **(B, C)**, Comparison of water loss rate of detached rosette leaves and roots from transgenic and wild-type plants. Plants grown under control and 2 weeks after drought stress were derooted and allowed to air-dry. Water loss was measured by recording the fresh weight (FW) of the shoots and roots 48 hours subsequent to detachment treatment. Error bars represent mean ± standard deviation of five biological replicates. **(D)** TEM observations of 4-week-old rosette leaves from 2 weeks drought treatment Col-0 and *UC16*. Red arrows display the chloroplast membrane, blue arrows display the plasma membrane. **(E)** The relative expression levels of the drought-responsive genes determined by qRT-PCR in 4-week-old rosette leaves of Col-0, EV, *UC16*, and *UC22* transgenic line plants. Error bars represent mean ± standard deviation of three biological replicates. Student`s t-test, compared to Col-0 Control. *P < 0.05, **P < 0.01. *ns*, statistically nonsignificant; Student’s t-test. Scale bars represent 1 μm and 200 nm **(D)**.

To explore the possible involvement of *UcFatB* in the response to stress stimuli, the *UcFatB*-OE plants were subjected to drought stress by withholding water. After 2 weeks of drought stress, no significant differences were observed in the relative water content in the roots of the normal growth and drought stress groups in both the transgenic (*UC16* and *UC22*) and wild-type plants. However, aboveground water loss in the shoots of *UC16* and *UC22* was reduced, resulting in decreased leaf chlorosis and wilting compared to wild-type plants (**Figures 5A–C**). Consistent with the drought stress-related phenotypes, the expression levels of dehydration-induced genes, including *RD29A*, *COR15A*, and *KIN1* (Yu et al., 2008; Wang et al., 2011), were constitutively higher in *UcFatB*-OE plants compared to wild-type plants, with even higher expression in plants exposed to dehydration. Moreover, mRNA expression levels of *DREB1A*, *DREB2A*, and *DDF1* were significantly increased in *UC16* and *UC22* plants, while the expression level of *RD29B* maintained stability (**Figure 5E**), suggesting that ABA-independent pathway dominates the ABA-dependent pathway in the drought resistant phenotype of *UC16* and *UC22*, although it is likely that the both pathways are affected. The expression levels of critical enzyme-encoding genes involved in ABA biosynthesis including *NCED3*, *ABA2*, and *AAO3*, as well as enzyme-encoding genes involved in carotenoids biosynthesis including *PSY*, *FBN6*, and *PDS*. *NCED3*, *ABA2*, and *FBN6* are slightly increased in *UC16* and/or *UC22*, respectively. However, mRNA expression level of *AOO3* was down-regulated in *UcFatB* overexpression plants (**Supplementary Figure 11E**), suggesting that the drought resistance of *UC16* and *UC22* plants is not simply due to enhanced ABA-related drought response.

Non-targeted lipid analysis revealed that 50 of the 62 DAMs were closely related to the composition of plant cell membranes, including the major lipid classes in plant cell membranes (16 GPLs, 8 STs, 3 SLs) and 23 DGs, which constitute the structural backbone of GPLs (**Figure 3B**; **Supplementary Table 2**). TEM observations further revealed severe damage to the plasma membrane, loss of integrity of the plasma membrane-cell wall adhesion, and chloroplast deformation in the wild-type leaves after 14 days of drought. By contrast, *UC16* and *UC22* plants exhibited intact chloroplasts and recruitment of intracellular vesicles at plasma membrane damage sites in mesophyll cells (**Figure 5D**). These findings suggest that changes in plasma membrane components in *UcFatB*-OE plants play a role in restoring the plasma membrane and maintaining the cell integrity after drought.

Furthermore, cuticular wax, consisting of an amorphous mixture of VLCFAs and their derivatives, is a major component of the cuticle covering the aerial organs of plants (Samuels et al., 2008; Li et al., 2016). Consistent with the increased VLCFA content (**Figure 1D**) and expression levels of genes involved in wax biosynthesis and transportation (i.e., *ECERIFERUM 3* (*CER3*)) (**Supplementary Table 5**) (Rowland et al., 2007) in *UcFatB*-OE plants, SEM observations revealed a significant increase in the number of wax crystals on the stem and leaf surfaces of *UcFatB-*OE plants compared to wild-type plants (**Supplementary Figures 10B**; **11A, B**). Furthermore, a chlorophyll leaching assay using 4-week-old leaves demonstrated that *UC22* leaves lost chlorophyll at a slower rate than wild-type leaves, indicating reduced cuticular permeability in the *UcFatB*-OE line (**Figure 2I**; **Supplementary Figure 5J**), consistent with an earlier study (Qin et al., 2011). These findings indicate that the increased accumulation of cuticular wax in *UcFatB*-OE plants may also contribute to their enhanced drought tolerance.

### 3.6 *UcFatB* overexpression results in enhanced tolerance to water deprivation in tomato

**Figure 6:**
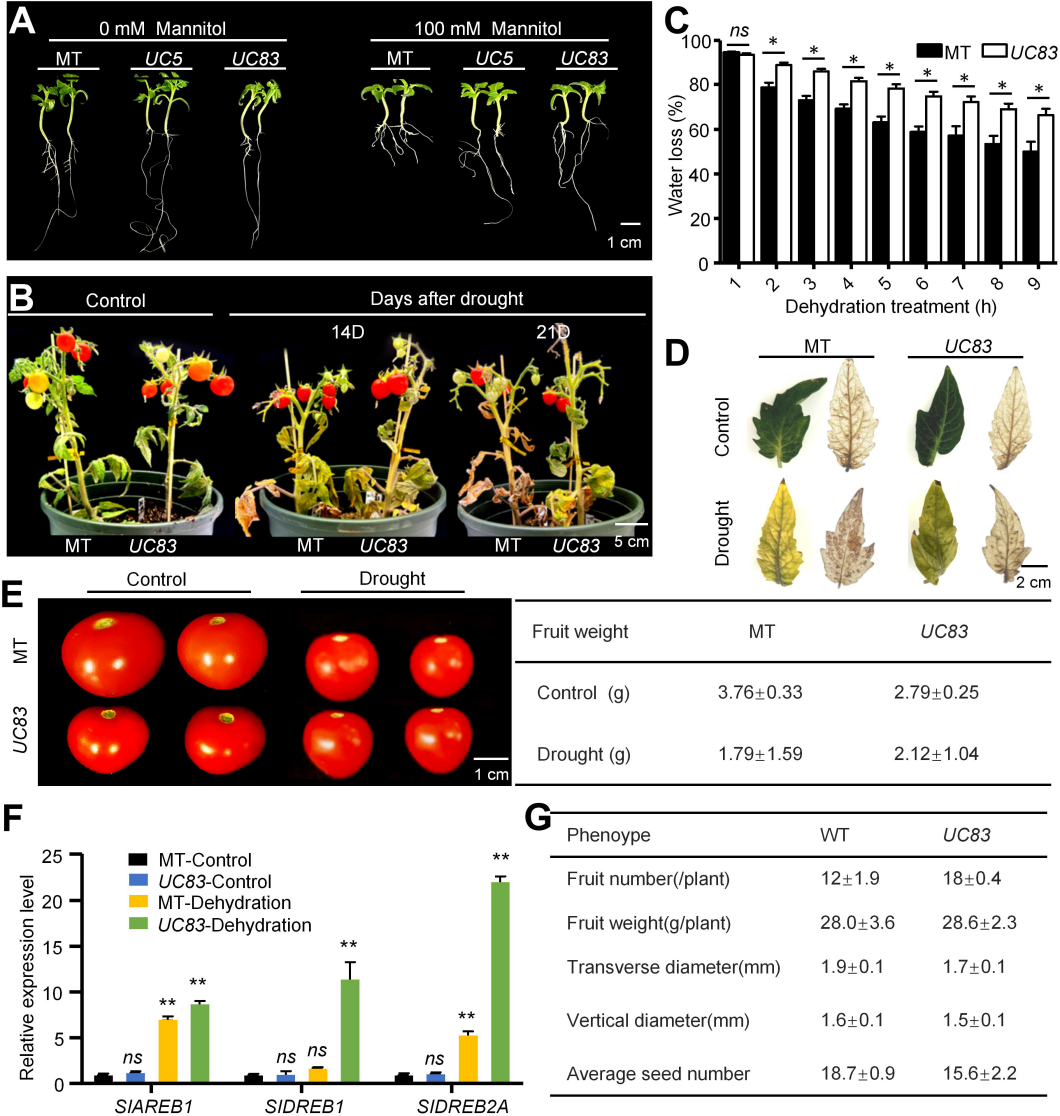
Ectopically expressed *UcFatB* and application of PCs in tomato confers tolerance to drought stress. **(A)** Phenotypic changes of 17 days seedlings in wild-type tomato cv. MicroTom (MT) and two independent *UcFatB*-OE plants (*UC5* and *UC83*) grown on 1/2 MS containing 0 and 100 mM mammitol. **(B)** Phenotypic changes of MT and *UC83* under well-watered control and drought stress conditions: 60-day-old plants were subjected to drought for 14 and 21 days. **(C)** Quantification of percent water loss in detached leaves of MT and *UC83* plants during drought treatment. Data represent the means ± standard deviation of three biological replicates. Student’s t-test, compared to MT. *P < 0.05. ns, statistically nonsignificant. **(D)** 3,3′-diaminobenzidine staining for reactive oxygen species (ROS) accumulation in leaves from MT and *UC83* plants exposed to drought treatment for 21 days. **(E)** Photograph (left) and quantification of per fruit (right) of MT and *UC83* under well-watered control and drought stress conditions. **(F)** Expression levels of the drought responsive genes determined by qRT-PCR in leaves of MicroTom and *UcFatB* overexpression plants. Data are means (± SD) of three biological replicates. Student’s t-test, compared to MT-Control. *P < 0.05, **P < 0.01. *ns*, statistically nonsignificant. **(G)** Quantification of fruit number, fruit size, fruit weight, and seed number in MT and *UC83* plants. Scale bars: 1 cm **(A)**, 5 cm **(B)**, 2cm **(D)**, and 1 cm **(E)**.

To further investigate the role of *UcFatB* in other drought-stressed plants, we generated *UcFatB-*OE tomato lines in the Micro-Tom (MT) background (**Supplementary Figures 12A, B**). Interestingly, the two independent T3-generation homozygous transgenic lines, *UC5* and *UC83*, exhibited normal development compared to the MT plants, including root growth and leaf morphology (**Figure 6A**; **Supplementary Figure 12C**). We further measured the fruit yield of *UC83* and MT lines grown in the greenhouse and found that the weight and seed number per fruit of the *UC83* plants was lower than that of the MT plants under normal conditions (**Figures 6E, G**). However, due to the significantly higher fruit number per plant of the *UC83* line compared to the MT line, the total yield per plant of *UC83* was slightly higher than that of MT (**Figure 6G**), suggesting that *UcFatB* overexpression does not have a negative impact on tomato yield.

Seven days post-germination in the MS culture medium, we examined the responses of *UC5* and *UC83* seedlings to osmotic stress after a 10-day mannitol (100 mM) treatment. We found that both *UC5* and *UC83* seedlings were less hypersensitive to mannitol compared to the MT plants, exhibiting fewer signs of deepened cotyledon color and reduced inhibition of root length (**Figure 6A**). In the breaker stage (~85 days post-germination), the *UC83* and MT plants were subjected to drought stress by withholding water. After 14 days of drought, the MT plants started to show leaf wilting, and this phenotype became more severe after 21 days of drought, whereas *UC83* transgenic plants showed less wilting and necrosis, with most leaves remaining viable (**Figure 6B**). Although the relative water content for all plants was decreased after drought, the rate of decline was slower in the OE than in MT leaves (**Figure 6C**). Furthermore, 3,3′-diaminobenzidine (DAB) staining showed that the cellular level of H_2_O_2_ was higher in MT leaves (14 days after drought) than that in *UC83* leaves (**Figure 6D**). Consistent with the drought stress phenotype, the expression levels of tomato dehydration-responsive genes, including *DREB1*, *DREB2A*, *AREB1*, and *CAT1*, were significantly induced in leaves of *UC83* than MT after the drought treatment (**Figure 6F**). Notably, the *UC83* plants produced larger fruits than the MT plants after 21 days of drought (**Figure 6G**). Collectively, our data suggest that *UcFatB* overexpression has a certain broad-spectrum effect on improving plant drought tolerance and has the potential to reduce yield loss in drought-stressed crops.

### 3.7 Exogenous application of methyl laurate and PCs alleviates drought stress in tomato

**Figure 7:**
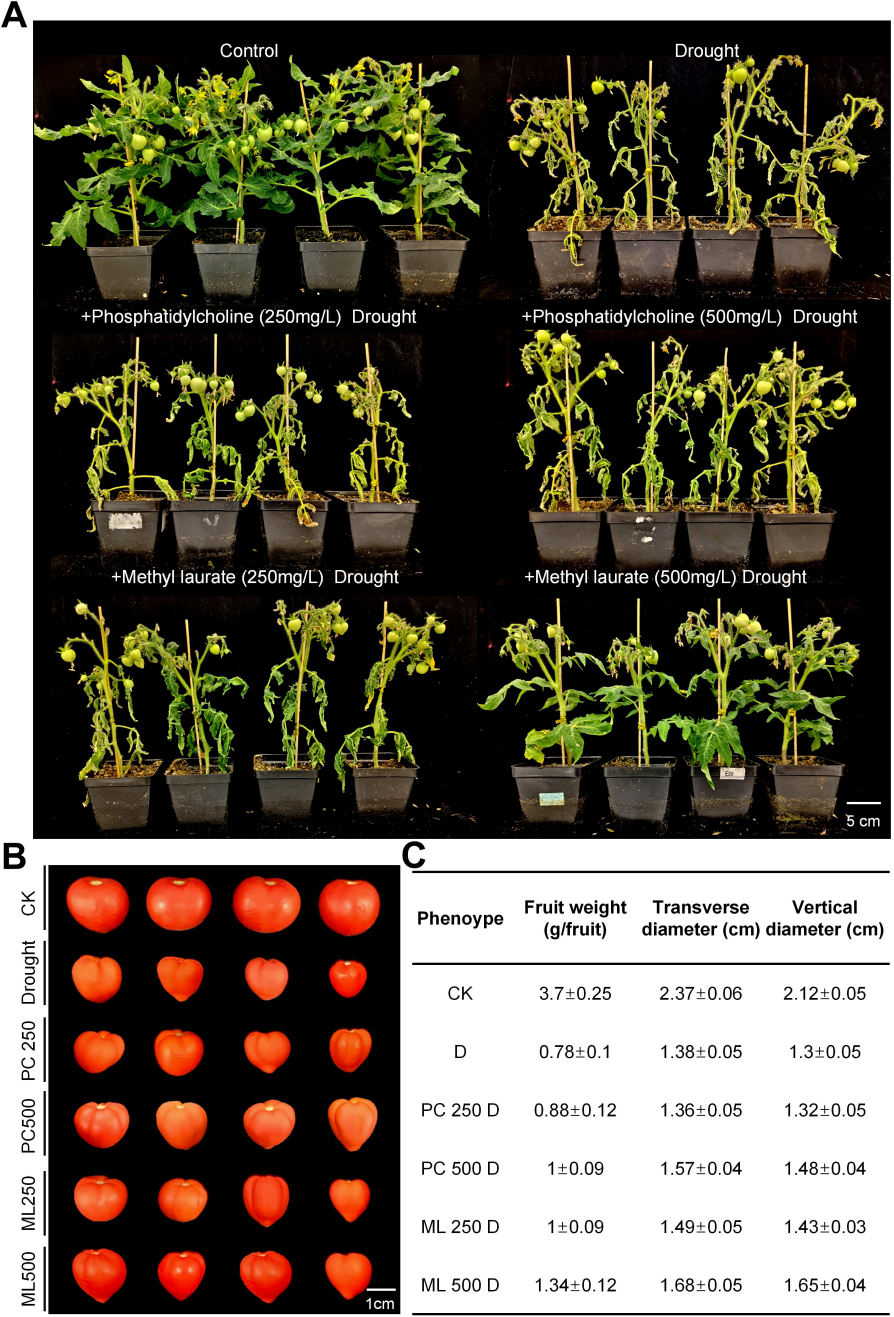
Application of methyl laurate and PCs in tomato confers tolerance to drought stress. **(A)** Phenotypic changes of MT plants under well-watered control and drought stress conditions: 45-day-old plants treated with exogenous methyl laurate (250 and 500 mg/L) and phosphatidylcholine (250 and 500 mg/L) were subjected to drought for 21 days. **(B, C)** Photograph **(B)**, size and weight quantification **(C)** of fruits produced by MT under well-watered control and drought stress conditions: 45-day-old plants treated with exogenous methyl laurate (250 and 500 mg/L) and phosphatidylcholine (250 and 500 mg/L) were subjected to drought for 21 days. Data represent the means ± standard deviation of three biological replicates.

Because of the low solubility and bioavailability of aqueous insoluble, lauric acid is hard to directly apply in the field of agriculture. Methyl laurate, the methyl ester derived from lauric acid, is an inexpensive, non-toxic and soluble compound often widely used in industrial, cosmetics and food fields. Our drought test showed that exogenous treatment of methyl laurate could significantly improve the drought tolerance of tomato plants. After 21 days of drought stress, wilting and necrosis in tomato plants pre-treated with exogenous methyl laurate (250 mg/L and 500 mg/L) were less severe than that in control-treated MT plants (~60 days post-germination) (**Figure 7A**).

Furthermore, non-targeted lipid analysis in *Arabidopsis* revealed that the total load of phosphatidylcholines (PCs) was increased by ~2.66-fold in *UC16* compared to wild-type Col-0 (**Figure 3C**; **Supplementary Table 2**), suggesting that PCs may improve the drought tolerance in *UcFatB*-OE plants. Consistent with our hypothesis, after 21 days of drought stress, wilting and necrosis in tomato plants pre-treated with exogenous PC (250 mg/L and 500 mg/L) effectively reduced the sensitivity of plants to drought compared with the control-treated MT plants (~60 days post-germination) (**Figure 7A**). Notably, exogenous application of both methyl laurate and PCs can eventually reduce the fruit size and weight loss caused by drought stress (**Figure 7B**). These findings suggest that as easily accessible and cost-effective chemicals, both methyl laurate and PCs have great potential for improving crop productivity in drought-prone areas.

## 4 Discussion

### 4.1 The accumulation of FAs in plants is strongly related to the substrate specificity of FATs

Acyl-ACP thioesterases (FAT) serve as housekeeping enzymes that provide cells with sufficient and specific chain-length supplies of FAs (predominantly 18:1 and 16:0) for plant growth and responses to environmental cues. MCFAs (C6-C12) and their derivatives are ideal feedstocks in nutraceutical, personal care, and oleochemical industries. However, the main MCFAs are manufactured from palm and coconut crops, which are restricted to tropical and subtropical regions, resulting in limited supplies and research studies in other higher land-plants (Kinsella et al., 2017). In *Cuphea palustvis*, *C. hookeriana*, *California bay*, and other oilseed plants, the middle-chain FAT coding genes are specifically expressed in the developing embryos and/or mature seeds, whereas the accumulation of MCFAs is strongly related to the substrate specificity of FATs *in vitro* and restricted to transgenic seed (Dehesh et al., 1996b, Dehesh et al., 1996a; Voelker, 1996). However, a subsequent study has shown that the overexpression of *Cuphea paucipetala FatB4* under the control of a seed-specific promoter induces widespread gene expression changes by altering the FA composition in *B. napus* transgenic seed (Nam et al., 2019). In addition, although expected to be 10:0/12:0-ACP-specific, two *Cuphea wrightii FatB* genes can produce a broad range of FAs (12:0, 14:0, 16:0) in *Arabidopsis* transgenic lines with the greatest accumulation of 14:0 FAs, suggesting that the substrate activity of FATs may undergo certain changes in different experimental systems, resulting in more developmental and/or environmental response-related impacts on plants.

### 4.2 MCFAs affect various plant growth processes, including seed germination, pollen exine formation, and leaf development

Previous studies in both *Arabidopsis* seeds and *Nicotiana benthamiana* leaves have indicated that the UcFatB enzyme displays the highest substrate specificity towards 12:0/14:0-ACP (Voelker et al., 1992; Reynolds et al., 2015). Consistent with these findings, fusing the *UcFatB* gene with the *AtFatB* promoter could not restore the reduced-size phenotype of *Arabidopsis fatb-1* leaves and seeds (**Figures 1A–C**), suggesting that substrate-specific FATs play distinct roles in plant development. In our study, the overexpression of the codon-optimized *UcFatB* gene driven by the *UBQ* promoter led to a higher substrate specificity toward 12:0-ACP rather than 14:0-ACP, whereas LCFAs (C16:0/16:1/18:0) and VLCFAs (C22:0/24:1) showed significant increases, resulting in the doubling of the total FA content in *Arabidopsis* rosettes (**Figure 1D**).

This contribution to the accumulation of FA metabolites negatively affected the equilibrium of normal plant growth processes, including seed germination, pollen exine formation, and leaf development (**Figures 2C–G**; **Supplementary Figures 3E, F**). In the case of pollen exine development, mutations of genes involved in lipid metabolism and transport that play important roles in the formation of the anther subcellular organelle membrane and pollen coat (tryphine) often cause microspore abortion and male sterility (Basnet et al., 2019; Chang et al., 2016). In *Arabidopsis*, *fatb-1* showed much smaller anthers containing fewer pollen grains than wild-type Col-0, whereas *UcFatB*-OE transgenic plants showed defects in pollen wall formation but not anther development (**Figures 2B,C**; **Supplementary Figure 3D**). Based on cytological examinations, molecular analyses, and electron microscopical observations, we confirmed the role of UcFatB in male infertility, in which *UcFatB* overexpression affected the assembly of the exine and pollen coat (**Figure 2C**; **Supplementary Figure 4F**). Given that *fatb-1* also displays multiple developmental changes, including reduced growth rate, fresh weight loss, wrinkled seeds, and reduced total FA content (Bonaventure et al., 2003), it is likely that the growth differences between *UcFatB-*OE and wild-type Col-0 plants caused by *UcFatB* overexpression generate the FAT paralog, which is highly expressed during development (**Figures 1A–C, E–G**, **2**). Interestingly, compared to *Arabidopsis*, the effects of *UcFatB* overexpression on tomato development are almost negligible, except for the weight and seed number per fruit under normal conditions (**Supplementary Figures 12C, D**), suggesting that the effects of MCFAs on plant development may be conserved in terms of fertility. Further studies are needed to determine the specific functions of MCFAs during plant development by including more plant receptor materials and employing additional metabolic analysis methods.

### 4.3 The enhanced drought tolerance in *UcFatB*-OE plants is partially due to the regulation of root architecture, wax deposition, and cell membrane integrity

The occurrence of frequent and extreme drought worldwide has substantially impacted crop growth and production. To cope with water deficiency, in addition to evolving complex morphologies and root systems to minimize drought stress, plants have developed the ability to resist low tissue-water content under drought stress, which involves osmotic regulation, cell elasticity, and epidermal wax formation (Batool et al., 2019). In combination analysis of correlations using transcriptomic and lipid metabolic data of *Arabidopsis* rosette leaves, most of the top 20 DEGs were associated with drought tolerance, and further analysis suggested that *UcFatB* overexpression leads to strong drought-resistant phenotypes in both *Arabidopsis* and tomato (**Figures 5**, **6**). In *Arabidopsis*, the root system of *UcFatB*-OE seedlings displayed reduced growth compared to wild-type seedlings, and *UcFatB* overexpression inhibited the sensitivity to root growth inhibition in response to mannitol treatment. These findings indicate that the architecture of the *UcFatB-*OE root system was modified to more efficiently absorb water from the soil surface by coordinating the elongation and/or the differentiation of root apex cells (**Figure 6A**; **Supplementary Figures 5B, C, E, F**), consistent with earlier studies (Passioura, 2002; Kim et al., 2015). Interestingly, the soil water content of the control and *UcFatB* overexpression plants was at a comparable level throughout the 20-day drought period (**Supplementary Figure S9A**), suggesting that these plants grew under similar drought conditions and *UcFatB* overexpression does not affect the water usage. The thicker waxy cutin coating and increased membrane integrity of *UcFatB*-OE leaves revealed that *UcFatB*-OE plants have also evolved strategies to withstand drought stress by adjusting osmotic processes within leaves and other major plant organs as well as improving antioxidant defenses (**Figure 5D**; **Supplementary Figures 11A, B**), consistent with previous studies (Turner, 1991; Batool et al., 2019). During drought spells, abscisic acid, the main hormone regulating the drought response in plants, triggers changes in stomatal behavior, root growth, gene expression, and cellular protection (Fang and Xiong, 2014). However, we found that abscisic acid induced stomatal closure in *UcFatB*-OE plants similar to that in wild-type plants (**Supplementary Figures 11C, D**). Taken collectively, our findings indicate that the enhanced drought tolerance in *UcFatB*-OE plants is partially due to the regulation of root architecture, wax deposition, cell membrane integrity, and the increased expression of the key stress-resistance related transcription factor encoding genes.

### 4.4 *UcFatB* overexpression not only affects the lauric acid accumulation/signaling, but also induces the lipid profile perturbation

To confirm whether the overexpression of the allogenic gene *UcFatB* induces alterations in the lipid profile of *Arabidopsis*, we utilized 4-week-old rosette leaves to execute fatty acid components, transcriptomic, and metabolic analyses (**Figure 1D**). This result indicated that *UcFatB* not only instigated the synthesis of lauric acid but also engendered changes in longer-chain fatty acids. Additionally, via quantitative PCR analysis, we discerned that the expression levels of genes encoding key enzymes related to abscisic acid synthesis and carotene synthesis underwent certain alterations subsequent to *UcFatB* overexpression (**Supplementary Figure 11E**), which substantiated that *UcFatB* did induce lipid spectrum perturbation. Concerning the drought resistance phenotype, the exogenous application of methyl laurate can directly impact the drought resistance of plants (**Figure 7**), suggesting that the drought resistance phenotype effect of *UcFatB* overexpression is primarily a direct consequence of lauric acid accumulation/signaling. However, the perturbation of the lipid spectrum may trigger other developmental phenotypic variations. For instance, as a result of the change of ultra-long-chain fatty acid derivatives content, the sporopollenin synthesis related to the pollen exine formation of *UC16* and *UC22* plant pollen was increased (**Figure 2C**), culminating in a decrease in male fertility.

### 4.5 The exogenous application of methyl laurate and PC enhanced the resistance of tomato to drought stress

PCs are the main components of the plasma membrane in most eukaryotes and serve as a source of signaling molecules. They bind to membrane proteins to directly participate in membrane-mediated signal transduction. A previous study has demonstrated that the absorption of PC by peach can stabilize cell membrane structure, enhance membrane integrity, and improve tolerance to drought and salt stress (Sun et al., 2020). Similar to other drought-responsive genes, *UcFatB* overexpression causes growth deficits and yield losses in plants because *UcFatB*-related pathways are involved in many agriculturally-relevant traits such as seed size, cell elongation, and root development, and thus, it cannot be directly applied to agricultural production. In our study, the exogenous application of both methyl laurate and PC enhanced the resistance of tomato to drought stress (**Figure 7**), which could potentially be attributed to the maintenance of phospholipid homeostasis in the cell membrane by methyl laurate and PC. Compared to breeding and genetic modification methods for alleviating drought-stress-mediated damage, the utilization of methyl laurate and PC is a straightforward and convenient method that could potentially protect considerable human and financial resources.

## 5 Conclusion

In conclusion, our results provide insights into the identification and characterization of the *de novo* MCFA synthesis pathway and the negative effects of changes in plant metabolism, gene programming, and development. The drought resistance observed in *Arabidopsis* and tomato allows us to believe that the modification of the *UcFatB* gene or other middle-chain FAT coding genes is highly desirable in more crops if targeted in specific plant cell types, tissues, and/or organs to avoid unnecessary ectopic expression. In addition, compounds, such as methyl laurate and phospholipid metabolites like PCs, show enormous potential in the development of drought-resistant or other stress-tolerant crop varieties.

## Data Availability

RNA-seq data has been successfully deposited and made public. The accession numbers of our data are: SRR25905879, SRR25905876, SRR25905876, SRR25905874, SRR25905878, SRR25905875.

## Supplementary material

Supplementary Figure 1 Clustal W sequence alignment of UcFatB and other acyl-ACP thioesterase proteins from different plants.

Supplementary Figure 2 Phylogenetic tree of acyl-ACP thioesterase B gene family.

Supplementary Figure 3
*UcFatB* could not rescue the defective development phenotype of *Arabidopsis fatb-1*.

Supplementary Figure 4 Phenotypic analyses of plant growth of *Arabidopsis* wild-type Col-0, EV control, and *UcFatB*-OE transgenic plants.

Supplementary Figure 5 Phenotypic analysis of *Arabidopsis* Wild-Type, EV control, and *UcFatB*-OE transgenic plant seedlings and rosettes.

Supplementary Figure 6 Analysis of metabolites in the 4-week-old rosette leaves of Col-0 and *UcFatB* OE line plants.

Supplementary Figure 7 Analysis of transcripts in the 4-week-old rosette leaves of the Col-0 and *UcFatB* OE line plants.

Supplementary Figure 8 mRNA expression of fatty-acid metabolism related genes in 4-week old wild type, EV, *UC16* and *UC22* rosettes.

Supplementary Figure 9 Four species of PC are enriched during KEGG metabolic pathways and classified as linoleic acid metabolism (ath00591), alpha-Linolenic acid metabolism (ath00592), and glycerophospholipid metabolism (ath00564).

Supplementary Figure 10
*UcFatB* overexpression does not affect the water usage.

Supplementary Figure 11 Phenotypic analyses of stems, leaves, and stomata of *Arabidopsis* Col-0, EV control, and *UcFatB*-OE transgenic plants.

Supplementary Figure 12 Generation of the *UcFatB*-OE transgenic tomato plants.

Supplementary Table 1 Metabolites information of total lipids analysis in Col-0 and *UcFatB*-OE (*UC16*).

Supplementary Table 2 Metabolites information of heatmap in **Figure 3C**.

Supplementary Table 3 Kyoto Encyclopedia of Genes and Genomes (KEGG) metabolic pathway enrichment.

Supplementary Table 4 Differential metabolite identification in *UC16* transgenic and wild type plants.

Supplementary Table 5 The 3196 genes that showed significantly differentially changed mRNA expression levels in *UC16*.

Supplementary Table 6 Correlation analysis between differential metabolites and differential genes.

Supplementary Table 7 Primer sequences used in this study.
