# Supplementary material for: Overexpression of Umellularia californica FatB thioesterase affects plant growth and lipid metabolome leading to improved drought tolerance in Arabidopsis and tomato
Source: Front Plant Sci. 2025 Jan 10;15:1446210. doi: 10.3389/fpls.2024.1446210 (PMC11757637; doi:10.3389/fpls.2024.1446210)
Supplement: Supplementary Figure 1 — Clustal W sequence alignment of UcFatB and other acyl-ACP thioesterase proteins from different plants. [file DataSheet1.docx]

**Supplemental materials**

**Figure S1.** Clustal W sequence alignment of UcFatB and other acyl-ACP thioesterase proteins from different plants.

**Figure S2.** Phylogenetic tree of acyl-ACP thioesterase B gene family.

**Figure S3.** *UcFatB* could not rescue the defective development phenotype of *Arabidopsis* *fatb-1*.

**Figure S4.** Phenotypic analyses of plant growth of *Arabidopsis* wild-type Col-0, EV control, and *UcFatB*-OE transgenic plants.

**Figure S5.** Phenotypic analysis of *Arabidopsis* Wild-Type, EV control, and *UcFatB*-OE transgenic plant seedlings and rosettes.

**Figure S6.** Analysis of metabolites in the 4-week-old rosette leaves of Col-0 and *UcFatB* OE line plants.

**Figure. S7.** Analysis of transcripts in the 4-week-old rosette leaves of the Col-0 and *UcFatB* OE line plants.

**Figure S8.** mRNA expression of fatty-acid metabolism related genes in 4-week old wild type, EV, *UC16* and *UC22* rosettes.

**Figure S9.** Four species of PC are enriched during KEGG metabolic pathways and classified as linoleic acid metabolism (ath00591), alpha-Linolenic acid metabolism (ath00592), and glycerophospholipid metabolism (ath00564).

**Figure S10.** *UcFatB* overexpression does not affect the water usage.

**Figure S11.** Phenotypic analyses of stems, leaves, and stomata of *Arabidopsis* Col-0, EV control, and *UcFatB*-OE transgenic plants.

**Figure S12.** Generation of the *UcFatB*-OE transgenic tomato plants.

**Table S1.** Metabolites information of total lipids analysis in Col-0 and *UcFatB*-OE (*UC16*).

**Table S2.** Metabolites information of heatmap in Figure 3c.

**Table S3.** Kyoto Encyclopedia of Genes and Genomes (KEGG) metabolic pathway enrichment.

**Table S4.** Differential metabolite identification in *UC16* transgenic and wild type plants.

**Table S5.** The 3196 genes that showed significantly differentially changed mRNA expression levels in *UC16*.

**Table S6.** Correlation analysis between differential metabolites and differential genes.

**Table S7.** Primer sequences used in this study.


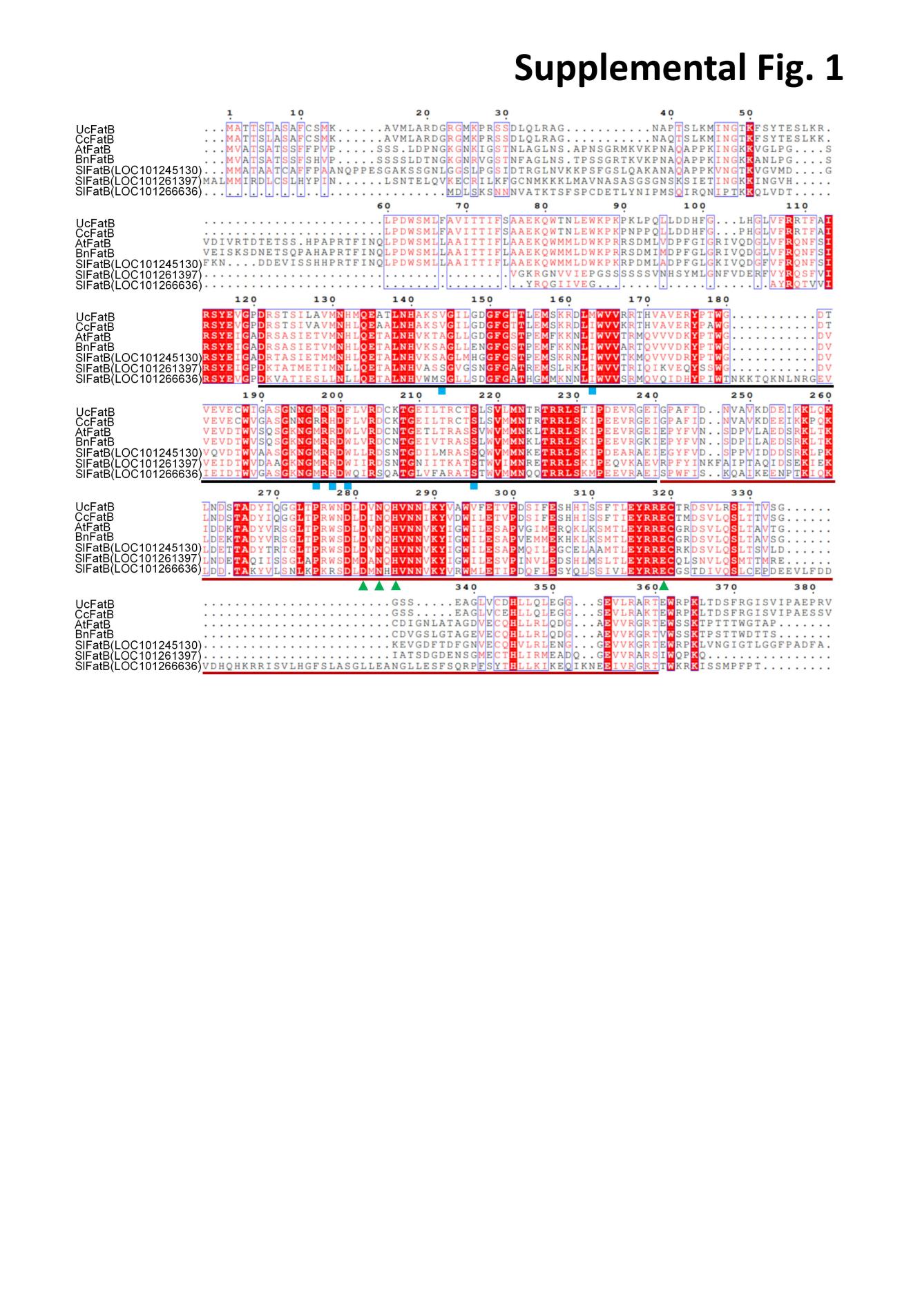
**Figure S1.** Clustal W sequence alignment of UcFatB with acyl-ACP thioesterases of different plants.

The residues highlighted by a bold black line above correspond to the N-terminal substrate binding domain contains the substrate binding pocket residues labeled with rectangles. The residues highlighted by a bold red line below correspond to the C-terminal catalysis domain contains the catalytic residues labeled with triangles. Red shading denotes conserved residues in all sequences.


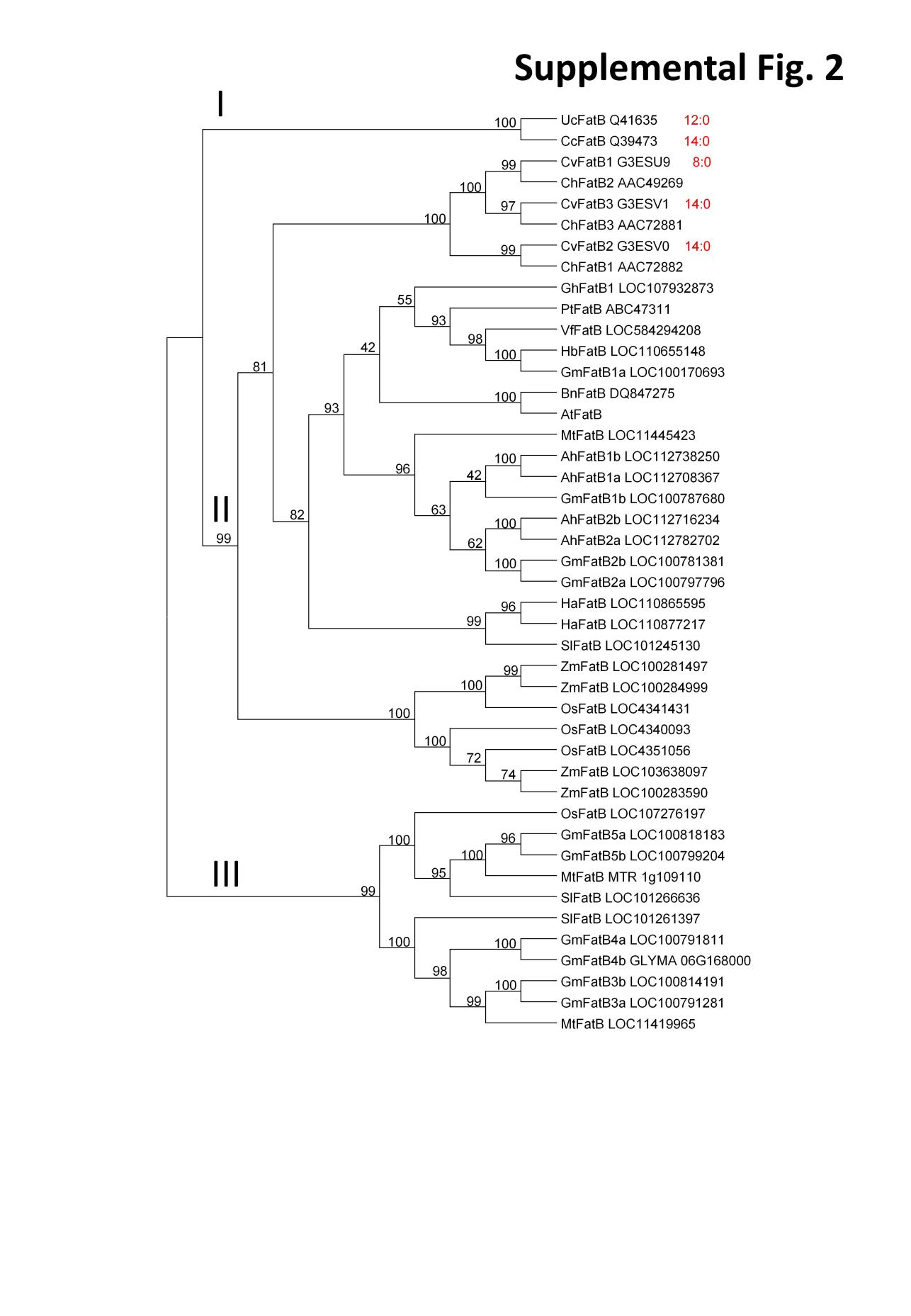


**Figure S2.** Phylogenetic tree of acyl-ACP thioesterases B gene family.

The protein sequences were subjected to a MUSCLE multiple alignment and phylogenetic tree was constructed by Neighbor-Joining (NJ) method using Mega IV. Bootstrap values for 1000 are shown on the branches. The name and abbreviation of plant species used for the analysis are Umbellularia californica (Uc); Arabidopsis thaliana (At); Cinnamomum camphora (Cc); Glycine max (Gm); Medicago truncatula (Mt); Brassica napus (Bn); Oriza sativa (Os); Vernicia fordii (Vf); Hevea brasiliensis (Hb); Solanum lycopersicum (Sl); Zea mays L (Zm); Arachis hypogaea(Ah); Cuphea hookeriana (Ch); Cuphea viscosissima (Cv); Populus tomentosa (Pt); Gossypium hirsutum (Gh). 8:0 refers to C8:0; 12:0 refers to C12:0; 14:0 refers to C14:0.

**
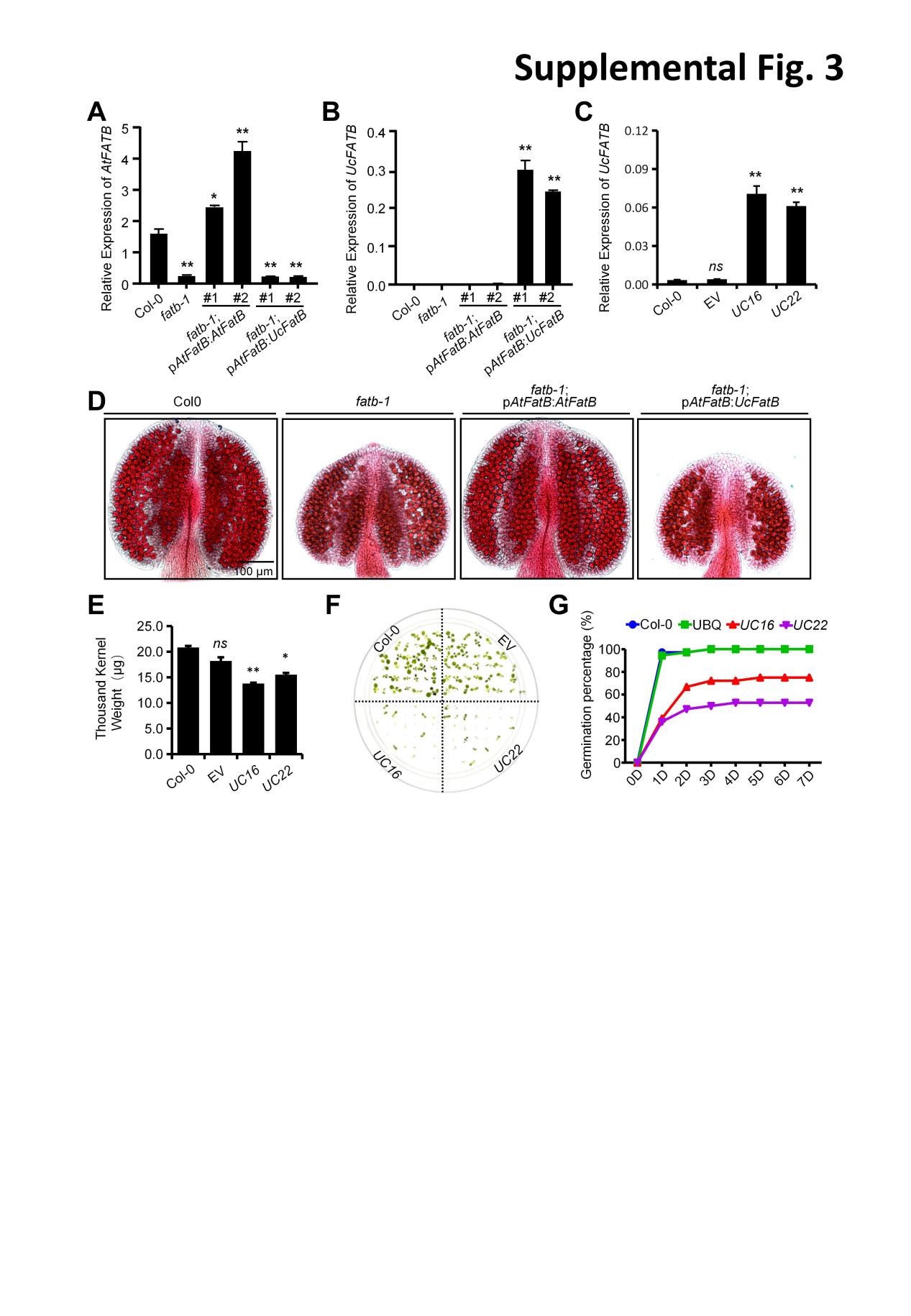
**

**Figure S3.** *UcFatB* could not rescue the defective development phenotype of *fatb-1*.

(A-B) Relative expression levels of *AtFATB* (A) and *UcFATB* (B) in wild-type, *fatb-1*, *fatb-1*; p*AtFatB*:*AtFatB*, and *fatb-1*;p*AtFatB*:*UcFatB* transgenic plants rosettes. *PP2C* was used as the control. (C) Relative expression levels of *UcFatB* in leaves of Col-0, empty vector-transformed control line, and *UcFatB* overexpression lines (*UC16*, *UC22*), respectively. *ACTIN* (*ACT2*) was used as the control. (D) Phenotypic analyses of anthers from wild-type, *fatb-1*, *fatb-1*; p*AtFatB*:*AtFatB*, and *fatb-1*; pAtFatB:UcFatB transgenic plants. (E) Seeds of wild type, EV, *UC16*, and *UC22* transgenic plants were dried properly, and 1000-seed weight were determined. (F) Seeds of wild type, EV, *UC16*, and *UC22* transgenic plants were allowed to germinate on 1/2 MS-agar plates and photographed 7 d after cold imbibition. Germination percentages were calculated. (G) Time course of seed germination frequencies in wild type, EV, *UC16*, and *UC22* transgenic plants. Dormant seeds were sown on 1/2 MS-agar plates and allowed to grown under long-day conditons. The germination frequency was measured daily. An average of triplicate experiments using a typical seed batch is presented with the standard deviation. Scale bars represent 100 μm. Three biological replicates were performed. Data are shown as the mean ± SD. P values were calculated by Student’s t-test. *, *P* < 0.05; **, *P* < 0.01. *ns*, statistically nonsignificant.


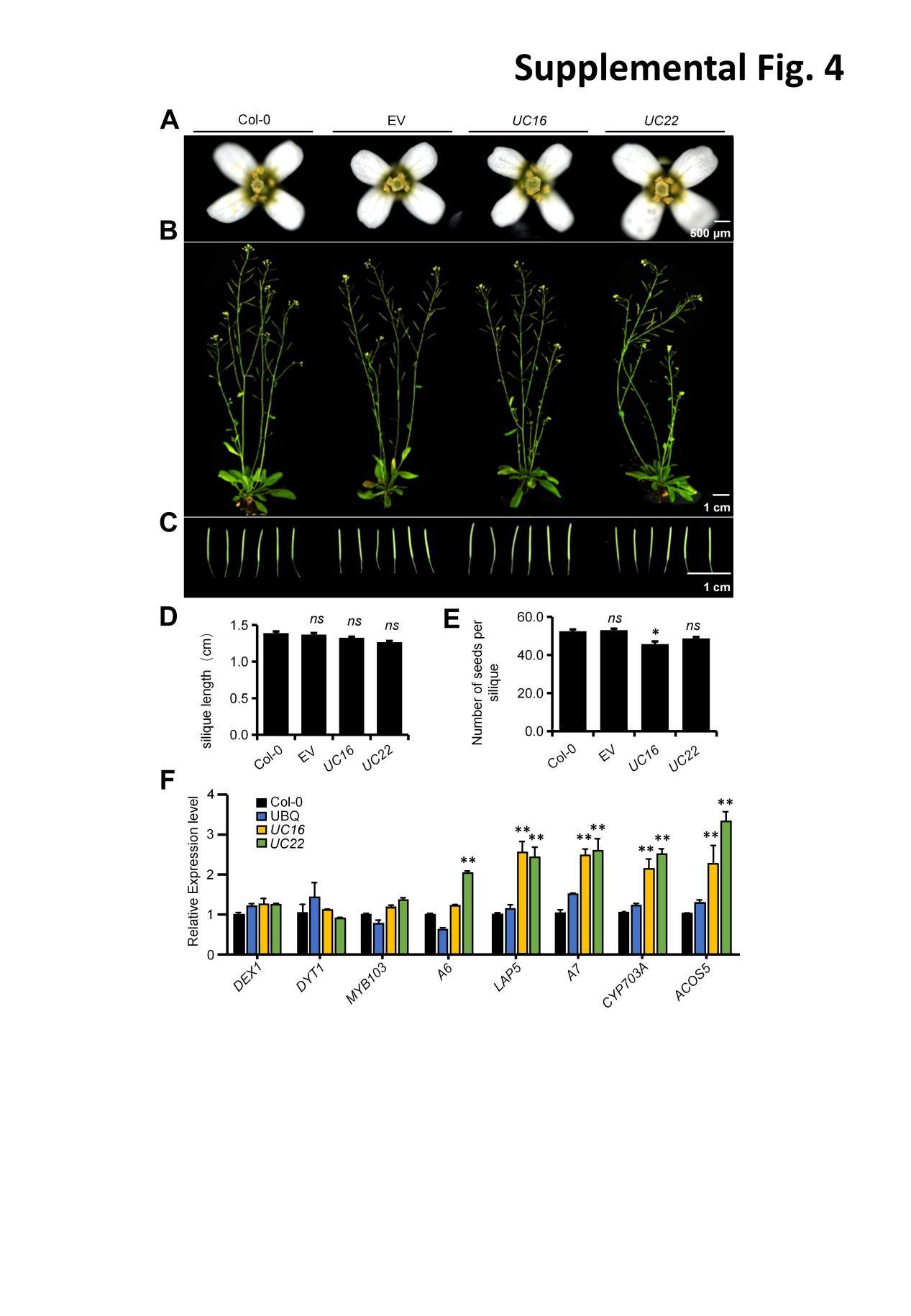


**Figure S4.** Phenotypic analyses of plant growth of Arabidopsis wild-type, EV control, and *UcFatB* OE transgenic plants.

(A-C) Phenotypic analyses of 6-week-old WT, EV control, and *UcFatB* OE flowers (A), plants (B), and siliques (C). (D-E) Analysis of silique length (D) and the number of seeds per silique of 6-week-old wild-type, EV, *UC16*, and *UC22* plants. (F) Quantitative RT-PCR results showing relative expression levels of tapetum and pollen exine marker genes in the inflorescences of *UC16*, *UC22* and corresponding control 6-week-old plants. Three biological replicates were performed. Data are shown as the mean ± SD. P values were calculated by Student’s t-test. *, *P* < 0.05; **, *P* < 0.01. *ns*, statistically nonsignificant.


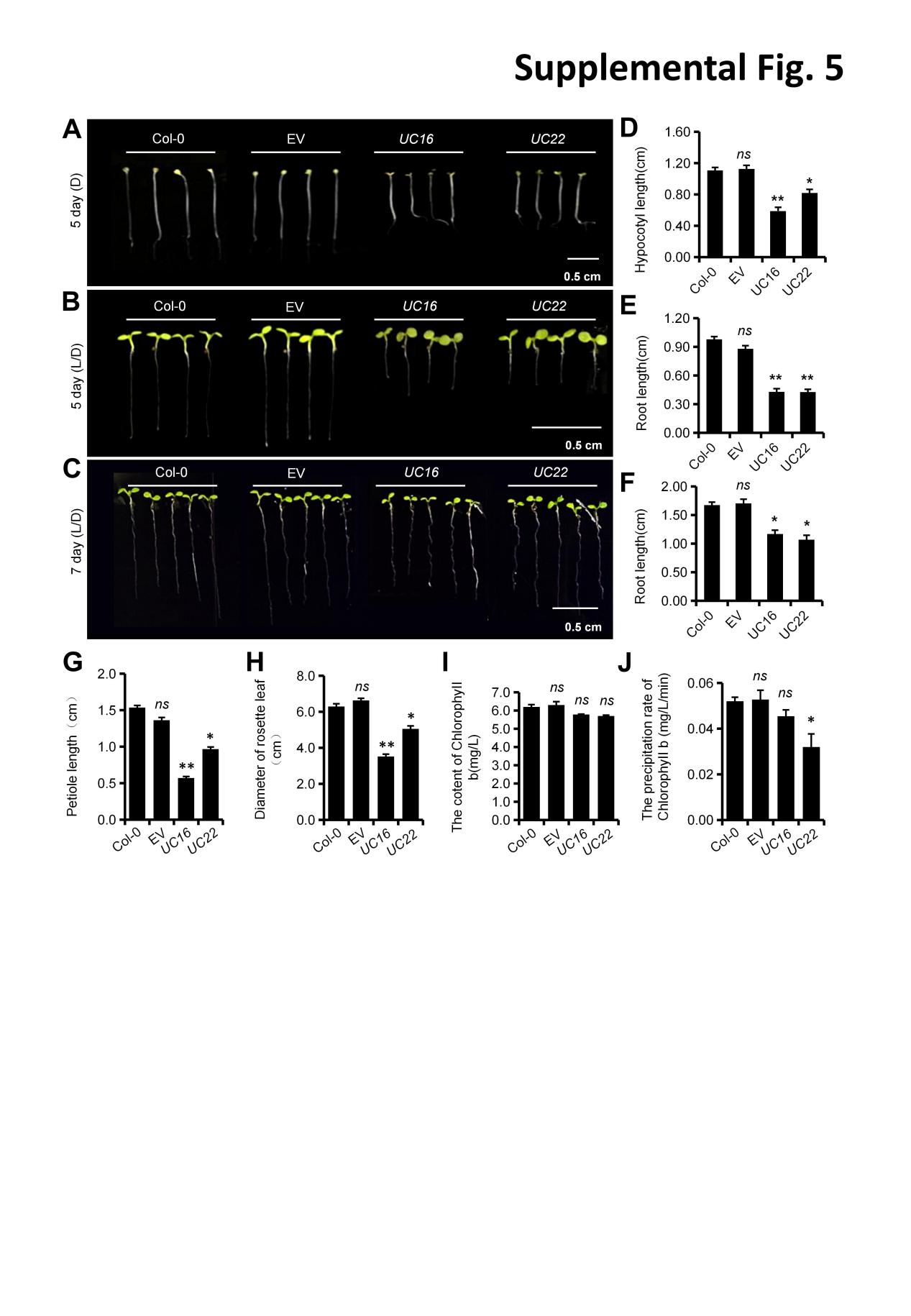


**Figure S5.** Phenotypic analysis of Arabidopsis Wild-Type, EV control, and *UcFatB* OE transgenic plant seedlings and rosettes.

(A) A phenotypic analysis of 5-day-old dark-grown WT, EV control, and *UcFatB* OE transgenic seedling hypocotyls. (B-C), A phenotypic analysis of 5-day-old (B), and 7-day-old (C) WT, EV control, and *UcFatB* OE transgenic seedling roots grown on 1/2 MS. (D-F) Analysis of hypocotyl length (D) and root length of 5-day-old (E), 7-day-old (F) wild-type, EV, *UC16*, and *UC22* seedlings. (G-H) Analysis of petiole length (G), diamiter (H) of leaves in 4-week old wild-type, EV, *UC16*, and *UC22* plants. (I) Analysis of the content of Chl b of the leaf from 4-week-old wild-type, EV, *UC16*, and *UC22* plants. (J) Chlorophyll b leaching from 4-week-old *UC1*6 and *UC22* transgenic plant rosette leaves. Three biological replicates were performed. Data are shown as the mean ± SD. P values were calculated by Student’s t-test, compared to Col-0. *, *P* < 0.05; **, *P* < 0.01. *ns*, statistically nonsignificant.

**
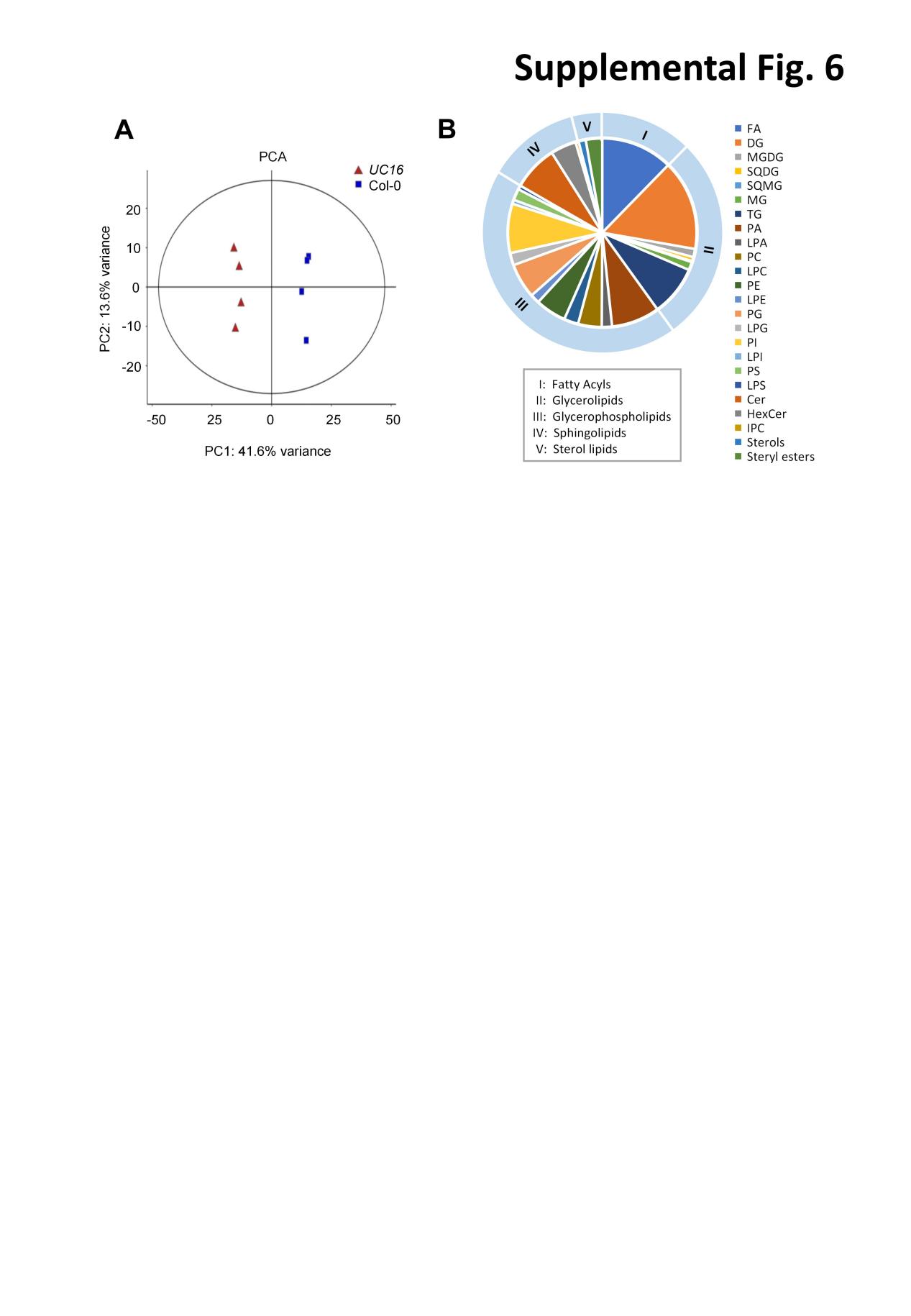
**

**Figure S6.** Analysis of metabolites in the 4-week-old rosette leaves of Col-0 and *UcFatB* OE line plants.

(A) Principal component analysis (PCA) of metabolome data. Four biological replicates per sample were analyzed for each genotype. (B) 569 species in 24 common classes of lipids validated in *UC16* and Col-0 leaves. The name and abbreviation of lipid species are Diglyceride (DG); Triglyceride (TG); Phosphatidylinositol (PI); Phosphatidic acid (PA); Ceramide (Cer); Phosphatidylglycerol (PG); Phosphatidylethanolamine (PE); Hexosyl ceramide (HexCer); Phosphatidylcholine (PC); Lyso-PC (LPC); Lyso-PG (LPG); Phosphatidylserine (PS); Lyso-PA (LPA); Lyso-PE (LPE); Monoglyceride (MG); Monogalactosyl diacylglycerol (MGDG); Lyso-PI (LPI); Lyso-PS (LPS); Sulfoquinovosyl diacylglycerol (SQDG) ; Ceramide phosphoinositols (IPC); Sulfoquinovosyl monoacylglycerol (SQMG).


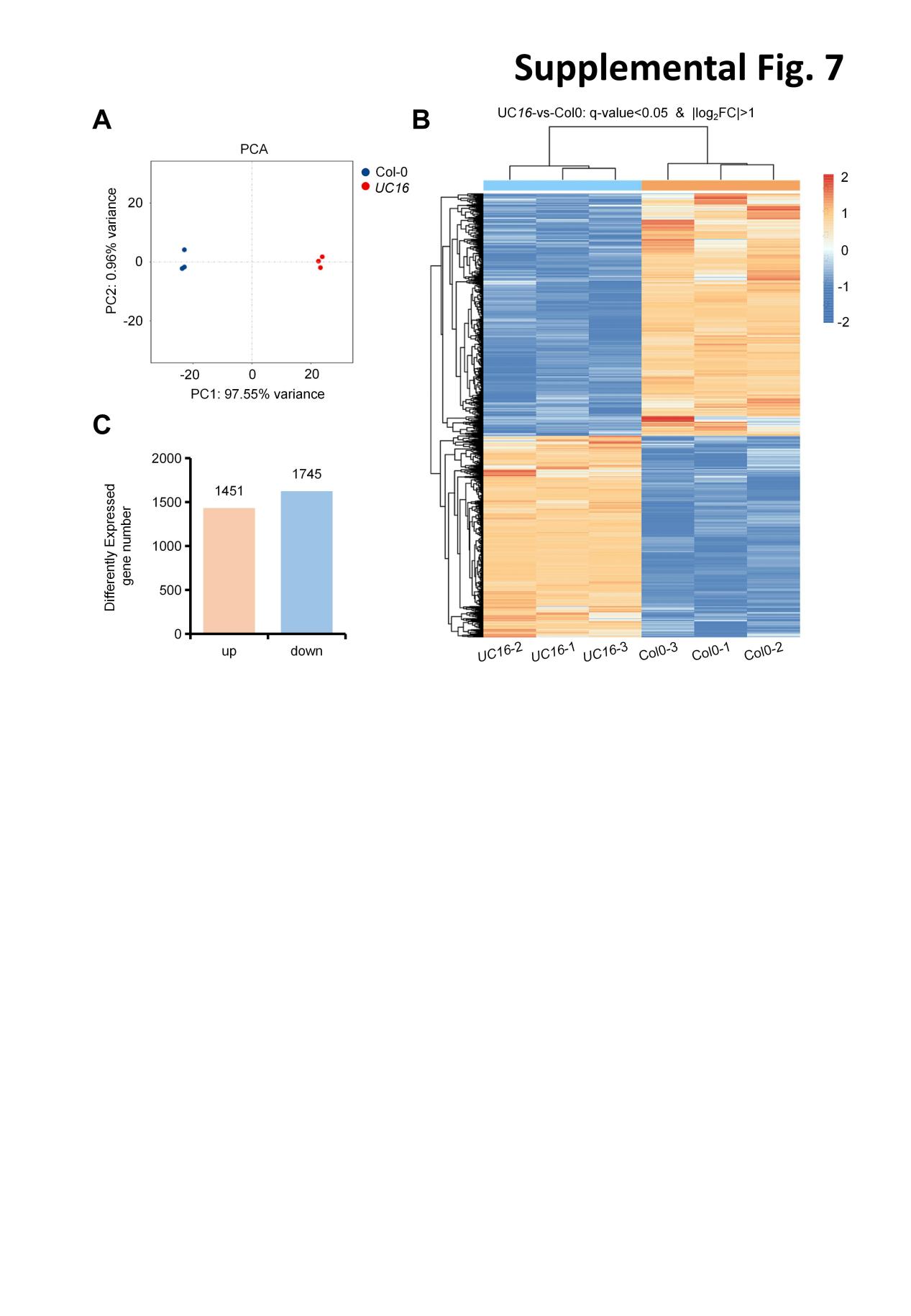


**Figure S7.**  Analysis of transcripts in the 4-week-old rosette leaves of the Col-0 and *UcFatB* OE line plants.

(A) Principal component analysis (PCA) of transcriptome data. Three biological replicates per sample were analyzed for each genotype. (B) Overall expression level log_2_FC of transcripts in the rosette leaves of the Col-0 and *UcFatB* OE line plants. (C) The number of significant differentially expressed genes up- or down-regulated (|log2FoldChange|>1; FDR adjusted p value < 0.05) in *UC16* vs. Col-0.

**
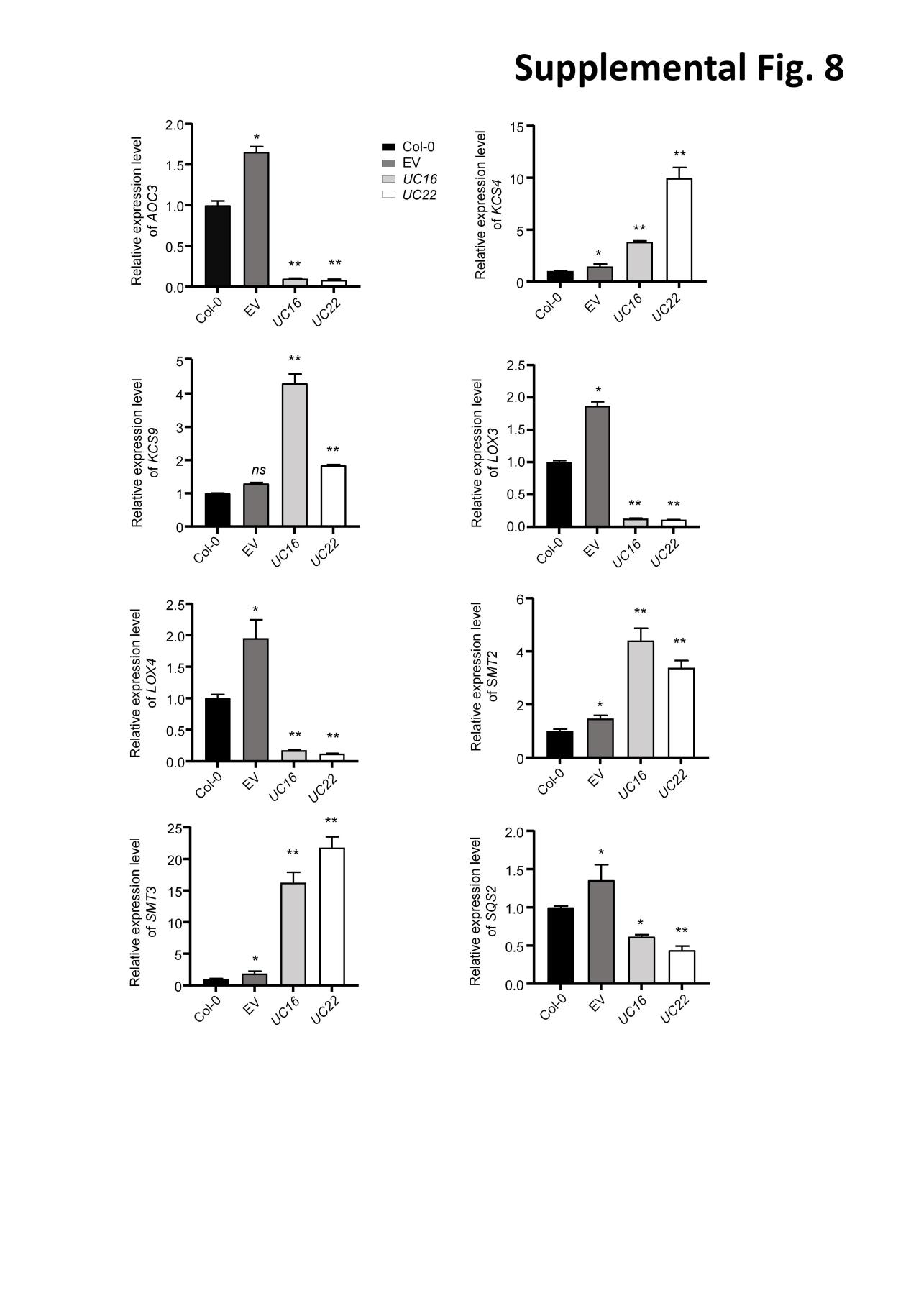
Figure S8.** mRNA expression of fatty-acid metabolism related genes in 4-week old wild type, EV, *UC16* and *UC22* rosettes.

*ACTIN* (*ACT2*) was used as the control. Three biological replicates were performed. Data are shown as the mean ± SD. *P* values were calculated by Student’s t-test, compared to Col-0. *, *P* < 0.05; **, *P* < 0.01.

**
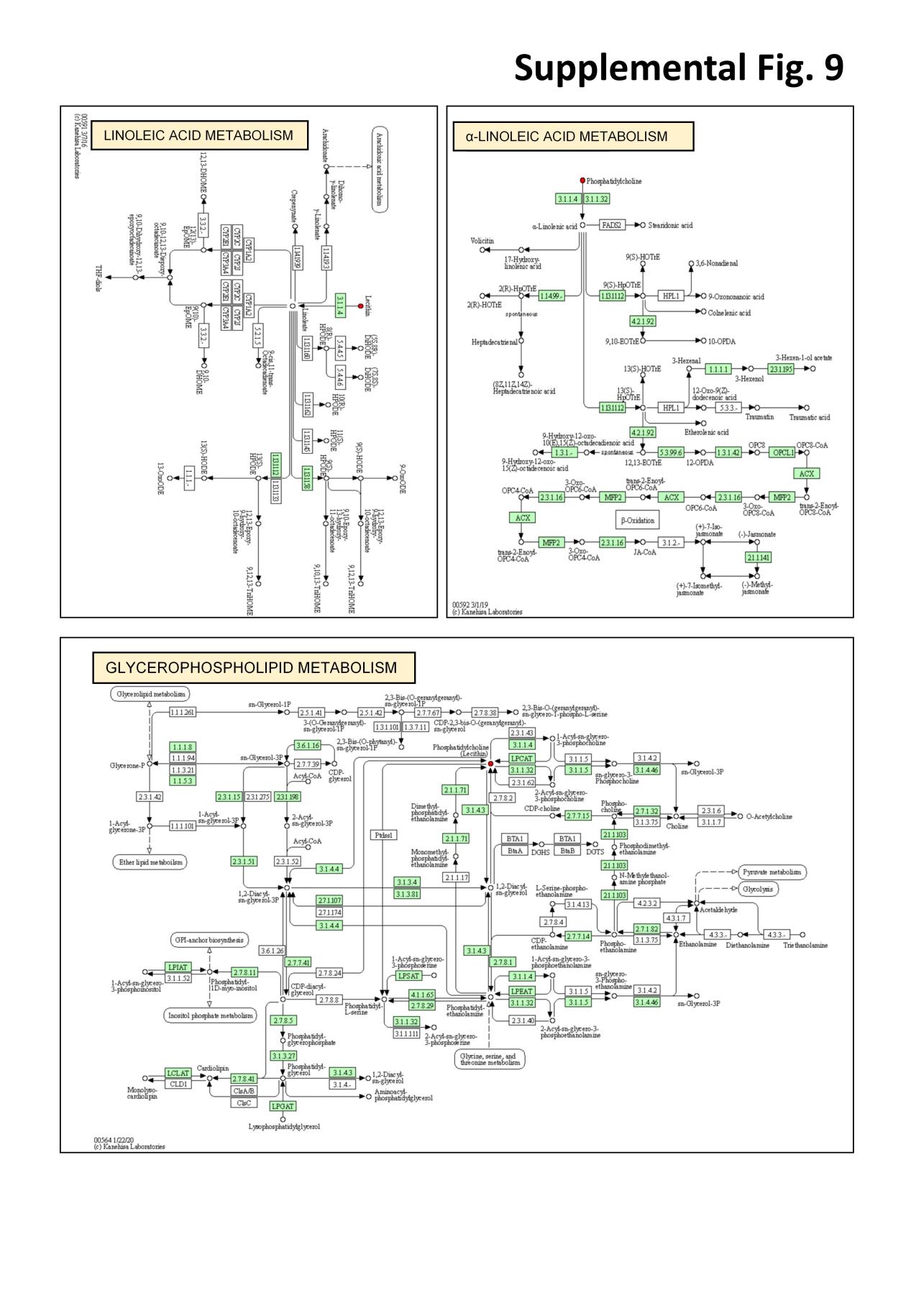
Figure S9.** Four species of PC are enriched during KEGG metabolic pathways and classified as linoleic acid metabolism (ath00591), alpha-Linolenic acid metabolism (ath00592), and glycerophospholipid metabolism (ath00564).

**
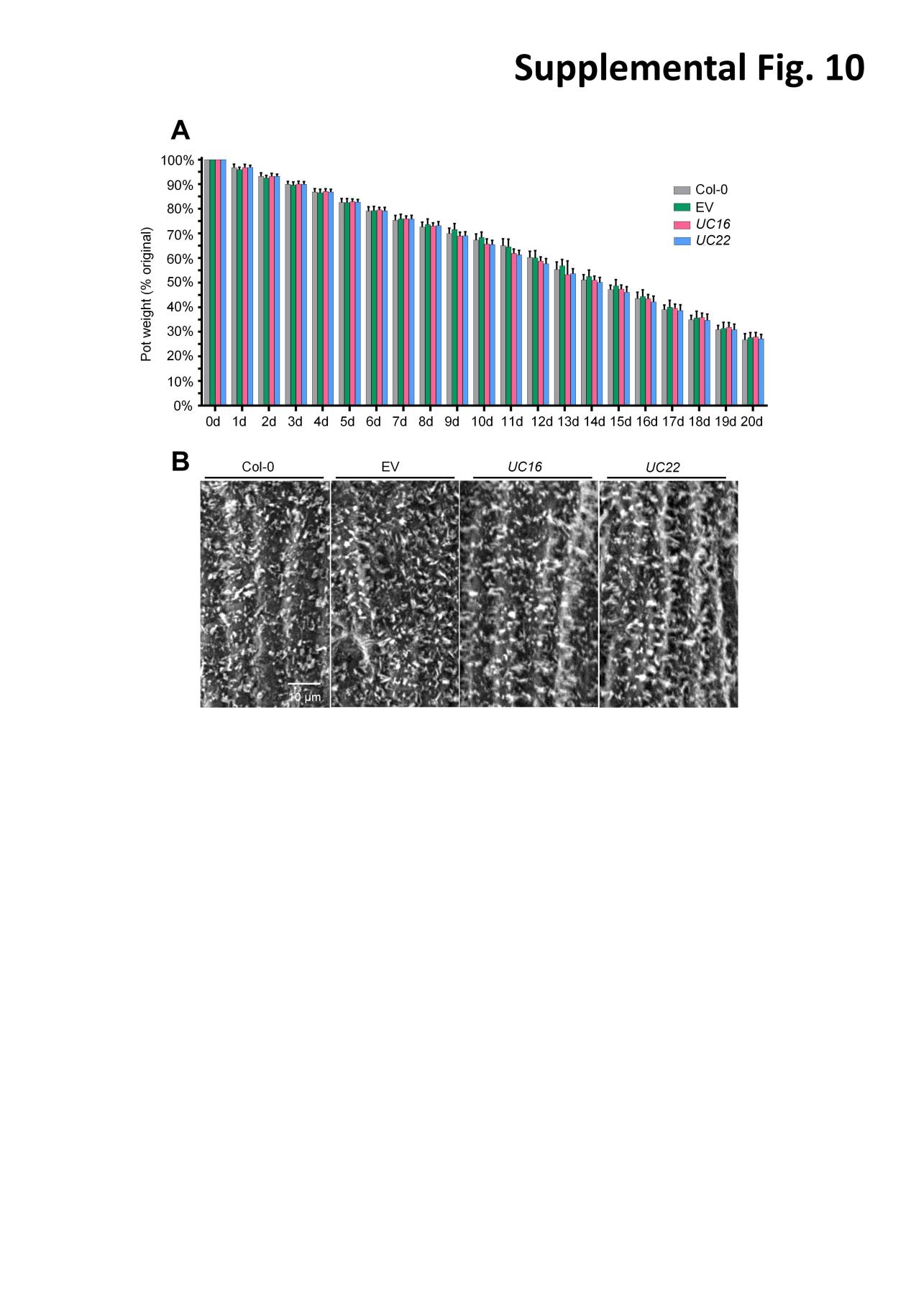
**

**Figure S10.** *UcFatB* overexpression does not affect the water usage.

1. Measurement of soil water content during drought. Measurements were taken from the first day of drought, during which the weight of the pots was measured daily for 20 consecutive days. The first day was recorded as 100%. Eighteen biological replicates were performed. Data are shown as the mean ± SD. *P* values were calculated by Student’s t-test, and the results are statistically nonsignificant. (B) SEM observations of stems from 6-week-old Col-0, EV, *UC16*, and *UC22* transgenic line plants. Scale bars: (A) 10 μm.

**
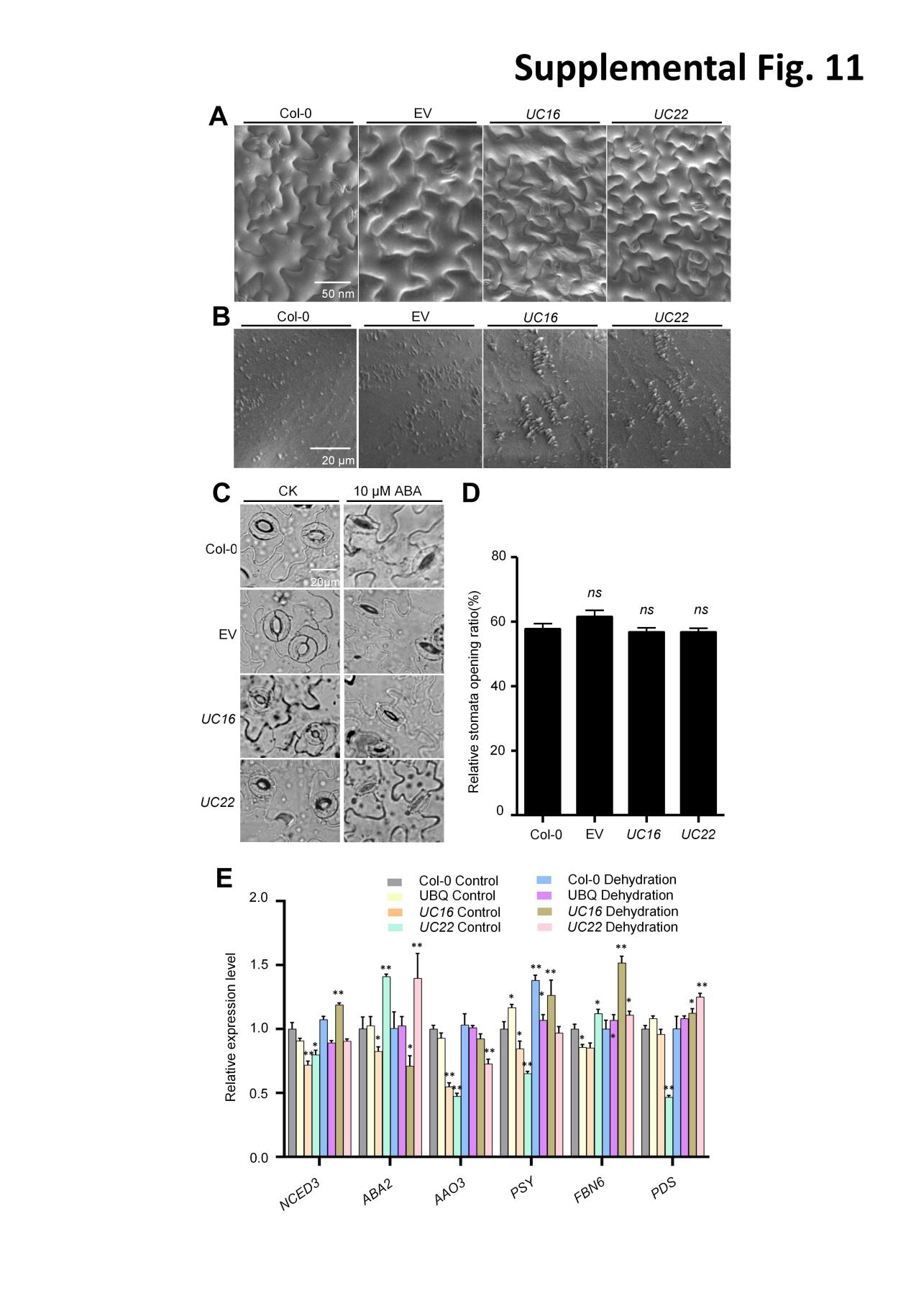
**

**Figure S11.** Phenotypic analyses of stems, leaves, and stomata of Arabidopsis wild-type, EV control, and *UcFatB* OE transgenic plants.

1. B) SEM observations of leaves from 6-week-old Col-0, EV, *UC16*, and *UC22* transgenic line plants. (C-D) Stomata and quantification of ABA induced stomatal closure in Col-0, EV, *UC16*, and *UC22* plants with or without 10 μM ABA treatment. Three biological replicates were performed. Data are shown as the mean ± SD (n ≥ 30). Student’s t-test, compared to Col-0. *, *P* < 0.05; **, *P* < 0.01. *ns*, statistically nonsignificant. (E) The relative expression levels of the ABA and carotenoids biosynthesis related genes determined by qRT-PCR in 4-week-old rosette leaves of Col-0, EV, *UC16*, and *UC22* transgenic line plants. Error bar represent mean ± standard deviation of three biological replicates. Student`s t-test, compared to Col-0 Control. **P* < 0.05, ***P* < 0.01. *ns*, statistically nonsignificant. Scale bars: (A) 50 nm, (B-C) 20 μm.


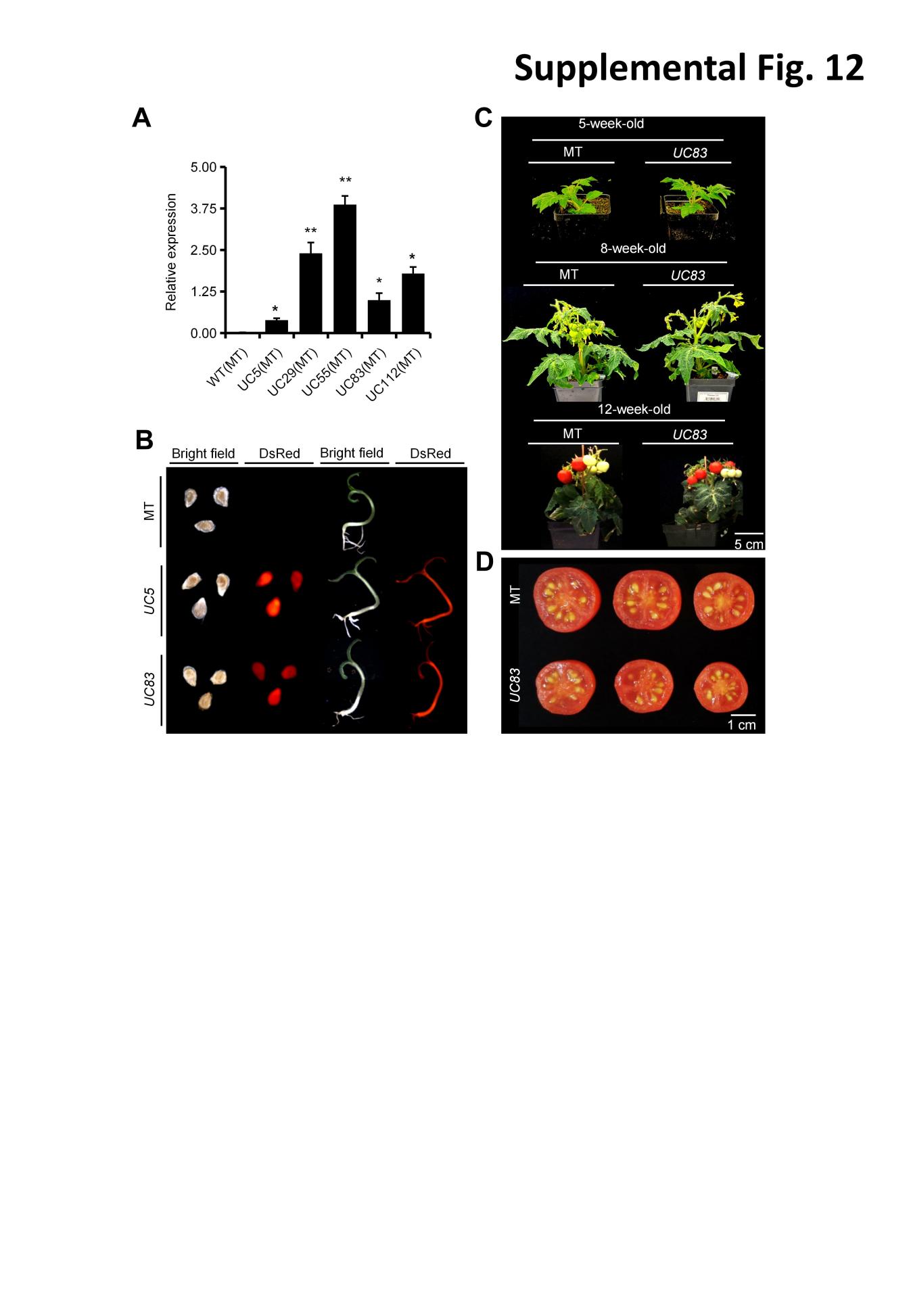


**Figure S12.** Generation of the *UcFatB* OE transgenic plants.

(A) Relative expression levels of *UcFatB* in leaves of MicroTom and *UcFatB* overexpression lines, respectively. Each data point represents mean ± SE (n ≥ 30), Student’s t-test, compared to MT. *, *P* < 0.05; **, *P* < 0.01. *ns*, statistically nonsignificant.(B) Flouoroscopic observations of the MicroTom and *UcFatB* overexpression lines. (C) Phenotypes of 5-week-old, 8-week-old, and 12-week-old MicroTom and *UcFatB* overexpression tomato plants. (D) Representative 52 dpa fruits of MicroTom and *UcFatB* overexpression plants (Bar = 1 cm).
